# Has gene expression neofunctionalization in the fire ant antennae contributed to queen discrimination behavior?

**DOI:** 10.1002/ece3.5748

**Published:** 2019-10-29

**Authors:** Viet‐Dai Dang, Amir B. Cohanim, Silvia Fontana, Eyal Privman, John Wang

**Affiliations:** ^1^ Biodiversity Research Center Academia Sinica Taipei Taiwan; ^2^ Biodiversity Taiwan International Graduate Program, Biodiversity Research Center Academia Sinica Taipei Taiwan; ^3^ Department of Life Science National Taiwan Normal University Taipei Taiwan; ^4^ Department of Zoology Southern Institute of Ecology Vietnam Academy of Science and Technology Hochiminh Vietnam; ^5^ Department of Evolutionary and Environmental Biology Institute of Evolution University of Haifa Haifa Israel

**Keywords:** fire ant, neofunctionalization, odorant‐binding protein, RNA‐Seq, *Solenopsis invicta*, worker antennae

## Abstract

Queen discrimination behavior in the fire ant *Solenopsis invicta* maintains its two types of societies: colonies with one (monogyne) or many (polygyne) queens, yet the underlying genetic mechanism is poorly understood. This behavior is controlled by two supergene alleles, *SB* and *Sb*, with ~600 genes. Polygyne workers, having either the *SB/SB* or *SB/Sb* genotype, accept additional *SB/Sb* queens into their colonies but kill *SB/SB* queens. In contrast, monogyne workers, all *SB/SB*, reject all additional queens regardless of genotype. Because the *SB* and *Sb* alleles have suppressed recombination, determining which genes within the supergene mediate this differential worker behavior is difficult. We hypothesized that the alternate worker genotypes sense queens differently because of the evolution of differential expression of key genes in their main sensory organ, the antennae. To identify such genes, we sequenced RNA from four replicates of pooled antennae from three classes of workers: monogyne *SB/SB*, polygyne *SB/SB*, and polygyne *SB/Sb*. We identified 81 differentially expressed protein‐coding genes with 13 encoding potential chemical metabolism or perception proteins. We focused on the two odorant perception genes: an odorant receptor *SiOR463* and an odorant‐binding protein *SiOBP12*. We found that *SiOR463* has been lost in the *Sb* genome. In contrast, *SiOBP12* has an *Sb*‐specific duplication, *SiOBP12b*′, which is expressed in the *SB/Sb* worker antennae, while both paralogs are expressed in the body. Comparisons with another fire ant species revealed that *SiOBP12b*′ antennal expression is specific to *S. invicta* and suggests that queen discrimination may have evolved, in part, through expression neofunctionalization.

## Graphical abstract

Queen discrimination behavior in the fire ant *Solenopsis invicta* maintains its two types of societies: colonies with one (monogyne) or many (polygyne) queens, yet the underlying genetic mechanism is poorly understood. We hypothesized that the alternate worker genotypes sense queens differently because of the evolution of differential expression of key genes in their main sensory organ, the antennae. We identified 81 differentially expressed genes using RNA sequencing, and follow‐up experiments revealed that antennal expression of an odorant binding protein, SiOBP12b', is specific to *S. invicta* and suggests that queen discrimination may have evolved, in part, through expression neofunctionalization.

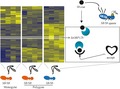

## 1 INTRODUCTION

Great variation has evolved in animal societies. Society complexity ranges from simple cooperative pairs (Pilakouta, Richardson, & Smiseth, 2015; Riehl, 2013) to huge but loose flocks and schools (Hemelrijk & Hildenbrandt, 2012), and even to the highly hierarchical colonies of ants, bees, and wasps (Hines, Hunt, O'Connor, Gillespie, & Cameron, 2007; Richards, Wettberg, & Rutgers, 2003; Taber, 2000). Variation can also be found intraspecies, such as in colony queen number in several ant species (Bourke & Franks, 1961; Hölldobler & Wilson, 1990). Although some studies exist (Kocher et al., 2018; Purcell, Brelsford, Wurm, Perrin, & Chapuisat, 2014), the evolutionary genetic bases underlying social form differences are still poorly understood. In particular, essentially nothing is known about whether differences in social communication genes can drive some of these social polymorphisms.

Social communication is a central feature of social insects and is mediated predominantly by chemical signals. Thus, the antennae, the major chemosensory organs in insects, play a critical role in chemical communication. Insect antennae are covered by many hair‐like structures called sensilla, which mediate the sensation of odor cues. These sensilla have pores on the cuticle that allows chemical molecules to enter into the aqueous sensillum lymph (Shanbhag, Müller, & Steinbrecht, 2000). Many biologically produced chemicals (semiochemicals) are hydrophobic and would have difficulty traversing the sensillum lymph to the chemoreceptors without help. Thus, the current model is that the semiochemicals are transported to chemoreceptors (i.e., odorant receptors (ORs), gustatory receptors, ionic receptors) by odorant‐binding proteins (OBPs) and chemosensory proteins (CSPs) (Reinhard, 2004). Consequently, the specificity of the OBPs and CSPs in the sensilla to a certain compound can directly affect insect behaviors (Matsuo, Sugaya, Yasukawa, Aigaki, & Fuyama, 2007; Pelletier, Guidolin, Syed, Cornel, & Leal, 2010).

A unique system for the study of chemical communication in social insects is queen acceptance or rejection behavior by workers in the red imported fire ant *S. invicta*. This case affords the investigation of the molecular genetic basis of behavior, because queen acceptance is completely linked to a social supergene with two alleles, *SB* and *Sb* (Keller & Ross, 1998; Wang et al., 2013). While the monogyne workers (all having the *SB/SB* genotype) accept only one *SB/SB* reproductive queen in their colonies, the polygyne workers (a mix of *SB/SB* and *SB/Sb* genotypes) accept multiple *SB/Sb* reproductive queens and kill all *SB/SB* queens. The queen discrimination behavior by the *SB/Sb* workers is presumably based on differences in cuticular chemical profiles between *SB/SB* and *SB/Sb* queens (Eliyahu, Ross, Haight, Keller, & Liebig, 2011; Gotzek & Ross, 2008; Ross & Keller, 1998; Trible & Ross, 2016). The identity of this cuticular chemical(s) that signals queen genotype is unknown; however, unsaturated hydrocarbons, polar biomolecules, and Gp‐9 have been proposed as candidates (Eliyahu et al., 2011; Gotzek & Ross, 2009; Trible & Ross, 2016). These findings indicated that there are at least two co‐evolved genes in this queen–worker interaction behavior: a chemical‐cue‐producing gene expressed in the queen and a chemical‐cue detection gene expressed in the worker. Although the initial studies found a link between the queen discrimination behavior and an OBP, *Gp‐9*, it is but one among ~600 protein‐coding genes within the supergene (Keller & Ross, 1998; Ross, 1997; Wang et al., 2013). Thus, which gene(s) underlie this queen discrimination behavior is still unknown and identification of the causative gene(s) is challenging, in part because recombination is suppressed within the supergene (Ross & Shoemaker, 2018; Wang et al., 2013).

In this study, we aimed to determine which genes are associated with the queen discrimination behavior in the fire ant workers. We reasoned that identifying the genes expressed in the worker antennae may reveal important genes underlying this behavior. However, there are other behaviors that are different between monogyne and polygyne workers beside the queen discrimination behavior, for example, higher intercolony aggression in monogyne compared to polygyne colonies. Therefore, distinguishing the genes that are regulated by genetic background or social environment is critical. Here, we used RNA sequencing (RNA‐Seq) to profile gene expression in the antennae of three worker classes: monogyne *SB/SB*, polygyne *SB/SB*, and polygyne *SB/Sb*. Given that odorant perception genes function in chemical recognition, we further examined two odorant perception genes that were differentially expressed between *SB/SB* and *SB/Sb* workers in a comparative genomic approach. Last, we compared gene expression across major body parts between two fire ant species to study whether high antennal gene expression for one odorant perception gene, *SiOBP12*, was conserved across species or specific to *SB/Sb* workers.

## 2 MATERIALS AND METHODS

Detailed methods are provided in the supplementary materials (Appendix S1).

### 2.1 Ant colony collection, maintenance, and genotyping

Colonies of *S. invicta* were collected from Taoyuan County, Taiwan. For *Gp‐9* genotyping, an PCR/RFLP assay was conducted on pooled DNA from at least ten workers (Krieger & Ross, 2002). Colonies were maintained under standard conditions (Jouvenaz, Allen, Banks, & Wojcik, 1977). Ants were sampled at least two to three months after collection from the field.

Two monogyne colonies of the tropical fire ant, *S. geminata*, were collected in Taichung City, Taiwan. After collection, we maintained them in the laboratory in the same conditions as *S. invicta*, except with the addition of seeds used as bird food.

### 2.2 Sample and tissue collection

All *S. invicta* and *S. geminata* samples were from foraging workers, which we obtained by attracting with honey. To minimize sampling differences, we selected only medium‐sized (ca. 1.5 mm in length) workers.

For *S. invicta*, we sampled different body parts from three classes of workers for each bioreplicate (Figure S1): monogyne *SB/SB* (M_BB), polygyne *SB/SB* (P_BB), and polygyne *SB/Sb* (P_Bb). For RNA sequencing, we extracted total RNA from 36 to 55 pairs of antennae for four bioreplicates (12 total samples). For qRT‐PCR experiments, we extracted total RNA from 20 to 24 antennal pairs or 15 units of a body part (head, thorax, or abdomen). For sequence trace analysis, we extracted total RNA from the antennae, heads, or thoraces–abdomens of 10–11 *SB/Sb* workers. Different experiments used different colony pairs.

Body part samples of *S. geminata* (head, thorax, and abdomen) were from 20 and 26 individuals. Antennae samples were from 99 and 108 individuals .

### 2.3 RNA extraction, amplification, and sequencing

We extracted RNA using the Direct‐zol RNA MiniPrep Kit (R2050, ZymoResearch) following a modification of the manufacturer's instructions (Zhang et al., 2013). To obtain sufficient cDNA for sequencing, we amplified each antennal RNA sample independently using the NuGEN Ovation V.2 kit (M01206, NuGEN Technologies), following the manufacturer's instructions. All samples were sequenced on the Illumina HiSeq 2500 platform with a 150 bp paired‐end protocol (at the High Throughput Genomics Core at the Biodiversity Research Center, Academia Sinica, Taiwan).

### 2.4 RNA‐Seq data processing

To generate an antennal reference gene list for differential gene expression analysis, we combined 15,589 genes from the fire ant official gene set (OGS) with novel transcripts within our RNA‐Seq data as follows. We first controlled the quality of the sequence reads of the 12 NuGEN amplified antennal cDNA libraries by retaining reads passing a base‐quality threshold of 20 and minimum length of 30 bases using cutadapt v1.16 (Martin, 2011). Next, we followed the Tuxedo pipeline (Trapnell et al., 2012) to map quality‐controlled reads onto the fire ant genome Si_gnH (Privman et al., 2018) and generated an expressed gene set containing 44,521 putative genes. We subsequently retained potential protein‐coding genes which encode peptides with an open reading frame of at least 50 amino acids and having similarity to genes in the NCBI nonredundant reference (Bethesda (MD)[Link]). This added 3,641 genes to the fire ant official gene set (Privman et al., 2018; Wurm et al., 2011), for a total of 19,230 putative protein‐coding genes in the reference gene set (OGS‐plus).

To identify differentially expressed genes (DEGs) on the full dataset of 12 samples, we first mapped the quality‐controlled reads onto OGS‐plus with Bowtie v2.2.6 (Langmead & Salzberg, 2012; Langmead, Wilks, Antonescu, & Charles, 2019). Next, we used RSEM v1.3.0 (Li & Dewey, 2011) to estimate gene expression levels from the mapped reads. Finally, we identified DEGs using the EBSeq package v1.2.0 (Leng et al., 2013).

### 2.5 Identification of the different paralogs of *SiOBP12* (*SiOBP12* and *SiOBP12b′*)

To obtain the cDNA sequence of the expressed *SiOBP12* in the *SB/Sb* worker antennae, we used RACE assays that were subsequently confirmed by cloning and Sanger sequencing. To estimate the expression of *SiOBP12* and *SiOBP12b*′, we mapped the merged quality‐controlled reads of the four bioreplicates of the *SB/Sb* antennal cDNA libraries onto the Si_gnH genome and used GATK for variant calling at the *SiOBP12* locus. To determine the expression of *SiOBP12* and *SiOBP12b*′ in the *SB/Sb* individual antennae, heads, and bodies (i.e., thorax–abdomen), we amplified partial cDNAs of the two paralogs using shared PCR primers and Sanger‐sequenced the PCR products without purification.

### 2.6 Structure analysis of SiOBP12 proteins

To examine whether the amino acid differences between *SiOBP12B*, *SiOBP12b*, *SiOBP12b*′, and *SgOBP12* may be potentially functionally important, we compared their predicted protein sequences to Gp‐9 (NCBI RefSeq Q8WP90) and the pheromone‐binding protein (PBP) in *Bombyx mori* (NCBI RefSeq NP_001037494). SiOBP12b′ was translated from RACE cDNA sequence obtained in this study (MN193778). SiOBP12B (Wurm et al., 2011), SiOBP12b (Wang et al., 2013), and SgOBP12 (Dryad) were translated from their respective genomic sequences. We aligned these six protein sequences using MEGA7 (Kumar, Stecher, & Tamura, 2016). The helices and the residues, which are likely to be involved in the bombykol binding pocket and pH‐sensitive conformation change, were adopted from previous studies (Krieger & Ross, 2005; Sandler, Nikonova, Leal, & Clardy, 2000).

### 2.7 Examination of *SiOR463* and *SiOBP12b′* in the *S. invicta SB, Sb,* and *S. geminata* genomes

To infer gene deletion or insertion, we examined the sequences of *SiOR463* and *SiOBP12b*′ and their surrounding regions in three PacBio genome assemblies: *S. invicta SB*, *Sb*, and *S. geminata*. The homologous sequences were aligned and visualized using Mauve (Darling, Mau, Blattner, & Perna, 2004). For *SiOR463*, we also mapped low‐coverage whole genome sequence of 14 males (Wang et al., 2013) onto the *S. invicta SB* and *Sb* contigs where *SiOR463* is located, and visualized mapping results in IGV (Thorvaldsdóttir, Robinson, & Mesirov, 2013).

## 3 RESULTS

### 3.1 Differentially expressed genes in the antennae of the three fire ant worker classes

To distinguish potential genetic (*SB/SB* vs. *SB/Sb*) or social (monogyne vs. polygyne) effects on antennal gene expression differences, we examined three worker classes: monogyne *SB/SB*, polygyne *SB/SB*, and polygyne *SB/Sb* (hereafter as M_BB, P_BB, and P_Bb, respectively, Figure S1A). We extracted and amplified total antennal RNA from four bioreplicates from each of the three worker classes for sequencing (12 total samples). RNA‐Seq yielded 47–61 million raw reads per sample, and after quality control, trimming, and filtering, 45–60 million reads were retained (see Appendix S1: Methods, Table S1). To identify differentially expressed genes (DEGs), we first estimated the expression level of each gene by mapping postfiltered reads onto “OGS‐plus,” a set of 19,230 putative protein‐coding genes, using Bowtie2 and counting the mapped reads with RSEM (Li & Dewey, 2011; see Appendix S1: Methods, Figure S1D). We used transcript sequences as the mapping reference (rather than the whole genome) to minimize the effect of transcribed noncoding regions (e.g., introns) on the mapping results.

**Figure 1:**
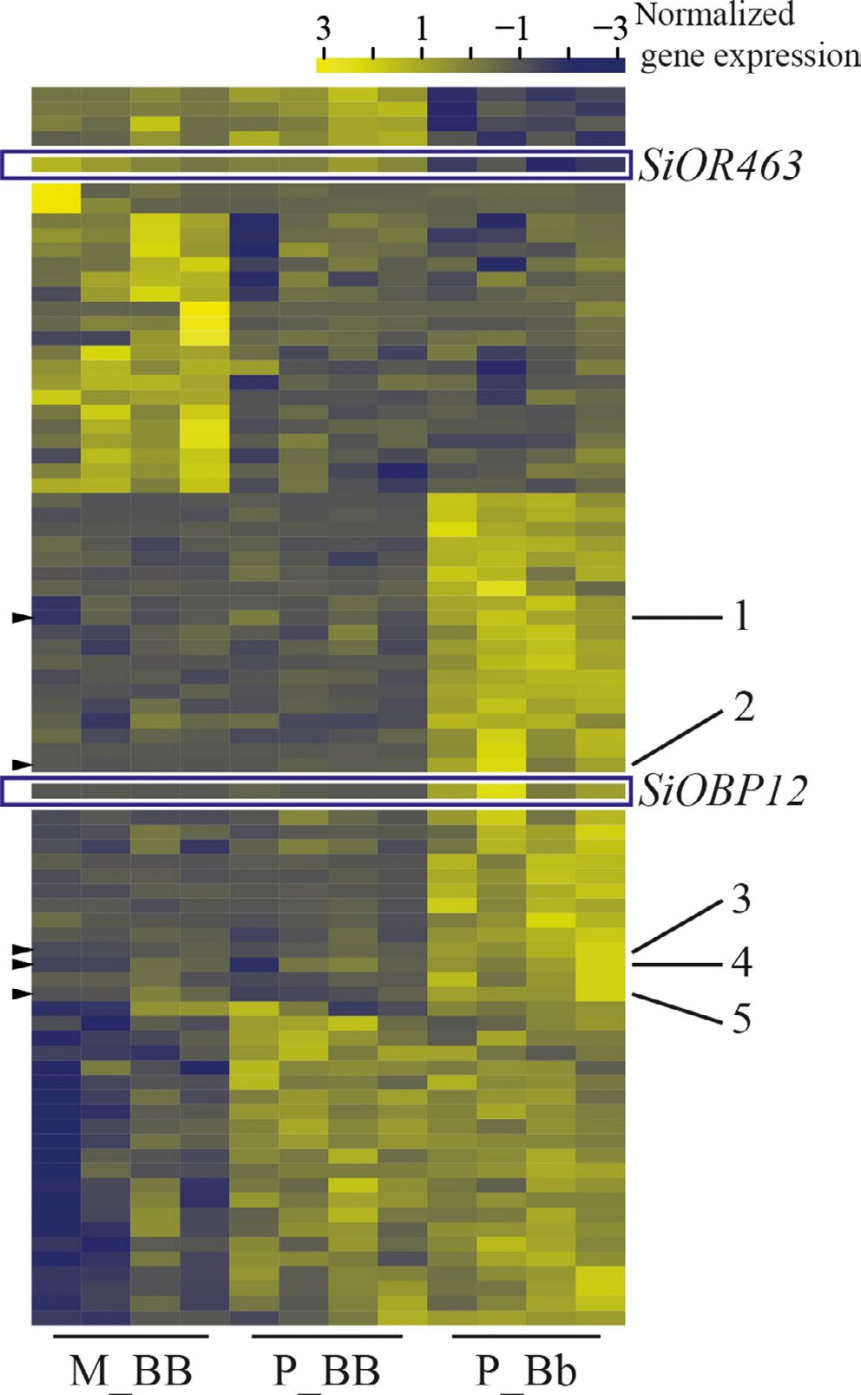
Heatmap of the 81 genes differentially expressed in the worker antennae in monogyne *SB/SB* (M_BB), polygyne *SB/SB* (P_BB), and polygyne *SB/Sb* (P_Bb). Gene expression was estimated by RSEM, using TPM (transcripts per million). Rows and columns represent genes and samples, respectively. Gene expression values are normalized by row. Genes located in the supergene are indicated with arrowheads and numbers (1. Uncharacterized protein, 2. Nadh dehydrogenase, 3. coiled‐coil domain‐containing protein 142, 4. tyrosine protein kinase transmembrane receptor ror, and 5. ubiquitin‐like domain‐containing ctd phosphatase) or boxes (*SiOBP12* and *SiOR463*). Gene order from top to bottom is the same as in Table S2

We identified 81 DEGs (posterior probability differentially expressed (PPDE) >95%; range 1.2‐ to 168‐fold difference; 46 with ≥2‐fold difference; Figure 1; Table S2). Of these, 36 genes were differentially expressed by social supergene genotype, with 31 and five genes being up‐ and downregulated, respectively, in P_Bb compared to both M_BB and P_BB. A comparison between social form samples revealed 43 genes differentially expressed with 21 and 22 being up‐ and down‐regulated, respectively, in monogyne (M_BB) compared to polygyne samples (P_BB and P_Bb). Two genes, *SINVm1_gene_00166* and *XLOC_023347*, were differentially expressed in all three comparison pairs (posterior probability of the expression pattern, >94%; PPDE >99%), possibly suggesting regulation by both genotype and social form. However, visual inspection and the Tukey HSD test suggested that genotype is the main factor regulating these two genes (Figure S2A, B). Definitive ascertainment of whether social form contributes to gene expression differences for these two genes will require greater sample sizes (Schurch et al., 2016).

The queen discrimination behavior in fire ants is linked to the social supergene (Keller & Ross, 1995; Wang et al., 2013). Of the 81 antennal DEGs, seven (8.64%) are located in the supergene and all were differentially expressed by genotype (Table S2). With respect to the 36 genes regulated by supergene genotype, this corresponds to a 6.68‐fold over‐representation of supergene genes (*p*‐value = .0002, Fisher's exact one‐tailed test). In contrast to a previous study on whole worker bodies, which found more genes regulated by social environment than by genotype (Wang, Ross, & Keller, 2008), we found a similar number of DEGs due to social environment (*n* = 43) and genetic effects (*n* = 38, including *SINVm1_gene_00166* and *XLOC_023347*) on the worker antennae (*p*‐value = .65, exact binomial two‐tailed test).

Inspection of the putative functions of all DEGs revealed 13 genes potentially involved in chemical metabolism (e.g., fatty acid synthase, cytochrome P450 enzymes, and esterase) and odorant perception (i.e., an OBP and an OR). Of these, eight genes were likely socially regulated with six and two genes being more and less expressed, respectively, in monogyne (M_BB) compared to polygyne (P_BB and P_Bb) samples. Five genes were regulated by genotype with four more highly expressed in *SB/Sb* (P_Bb) compared to *SB/SB* (M_BB and P_BB) samples.

Besides these two classes of genes, 16 transposable elements (TEs) were differentially expressed among the samples. Of these, the expression of six TEs was associated with social environment, with two and four TEs being more and less expressed, respectively, in M_BB samples compared to P_BB and P_Bb samples. Another ten TEs were differentially expressed by genotype, with two and eight (including *SINVm1_gene_00166*) genes being more and less expressed, respectively, in M_BB and P_BB compared to P_Bb samples.

**Figure 2:**
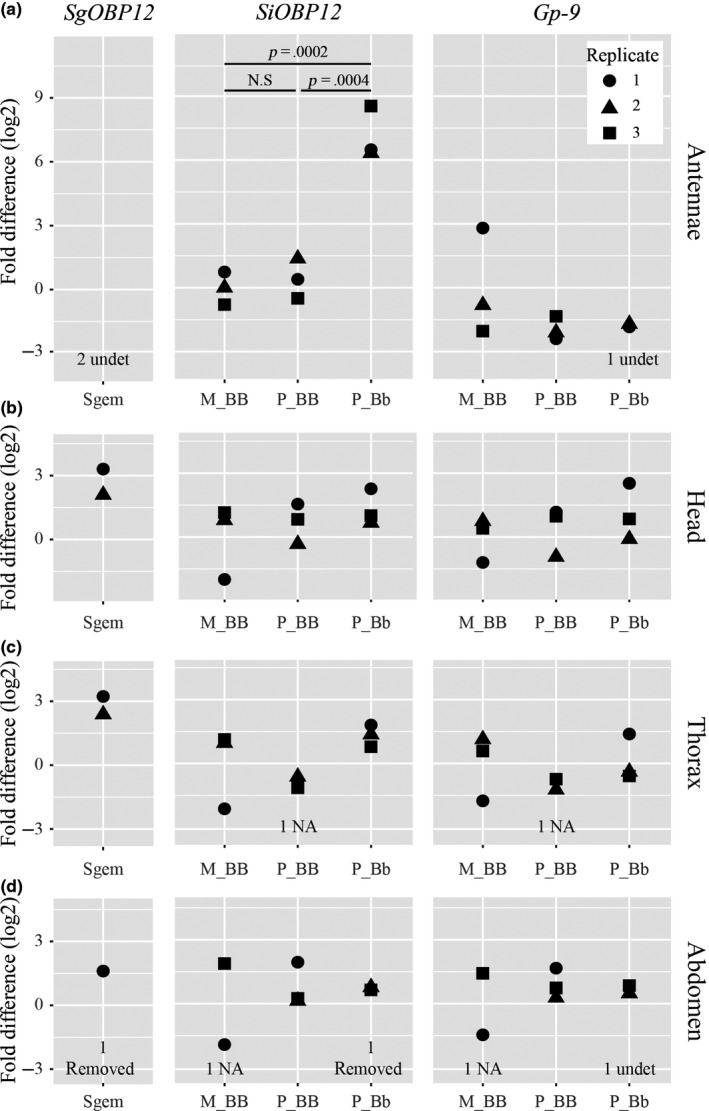
qRT‐PCR expression assays of *SgOBP12* (left), *SiOBP12* (middle), and *Gp‐9* (right) in different worker body parts. For *S. invicta* samples, each data point is the fold difference (log2) between the sample and the mean of the M_BB samples of the same gene in the same body part. *SiOBP12* and *Gp‐9* were not differentially expressed between the three worker classes in the worker heads, thoraces, and abdomens. In the worker antennae, only *SiOBP12* was highly expressed in P_Bb compared to both M_BB and P_BB. The expression of *SgOBP12* is relative to the M_BB samples of *SiOBP12*, that is, a fold difference value of 0 means that the expression level of *SgOBP12* relative to two internal controls (ΔCt) was equal to the average expression of *SiOBP12* relative to two orthologous internal controls (ΔCt) in M_BB in the same body part. In the head, thorax, and abdomen, the expression of *SgOBP12* was similar to that of *SiOBP12* in M_BB. Differential expression of the genes in different body parts was tested using the Tukey HSD test. Not shown *p*‐values are larger than 0.1. This assay cannot distinguish between the different alleles and paralogs of *SiOBP12*. The *S. invicta* head, thorax, and abdomen samples came from the same set of workers; the antennae samples were prepared independently. Data from the same bioreplicates of *S. invicta* are indicated with the same point shape. Each experiment consisted of three (*S. invicta*) or two (*S. geminata*) bioreplicates. Missing data points are due to low expression (undet), data removed for technical reasons (removed), or no assay for low RNA concentration (NA). Sgem, *S. geminata*; M_BB, monogyne *SB/SB*; P_BB, polygyne *SB/SB*; and P_Bb, polygyne *SB/Sb*

To verify the RNA‐Seq analysis, we conducted quantitative real‐time PCR (qRT‐PCR) assays on independent antennal RNA samples for four (of the seven) DEGs that are located in the supergene. We found the upregulation of all four genes in P_Bb samples compared to both M_BB and P_BB samples, confirming the RNA‐Seq analysis results (Figure 2a; Figure S3). We were unable to design specific qRT‐PCR primers for the remaining three genes.

### 3.2 The differentially expressed odorant perception genes have copy number variation

An OR *(XLOC_016888*, hereafter *SiOR463)* and an OBP (*SINVm1_gene_02111;* also known as *SiOBP12* (Gotzek, Robertson, Wurm, & Shoemaker, 2011)) were among the seven DEGs located within the supergene. OBPs and ORs are involved in the very first step of odorant perception, where a chemical is carried by an OBP (or a CSP) to a chemoreceptor and thus activating signal transmission (Leal, 2013). Because of a potential role in explaining the queen discrimination behavior, we analyzed these two genes in greater detail.

We identified *SiOR463* as a newly annotated *SB*‐specific OR gene, which has 95% nucleotide similarity to *SiOR163* (NCBI RefSeq XM_026133769.1), based on a new PacBio assembly of the *SB* genome. *SiOR463* is located between *SiOR163* and an unannotated OR, *SiOR462* (*SINVm1_gene_13698*). *SiOR463* has been deleted in the *Sb* genome (Figures S4–S7); thus, the approximate half expression level of *SiOR463* in *SB/Sb* compared to *SB/SB* individuals may simply reflect gene dose (Figure S2C).

**Figure 3:**
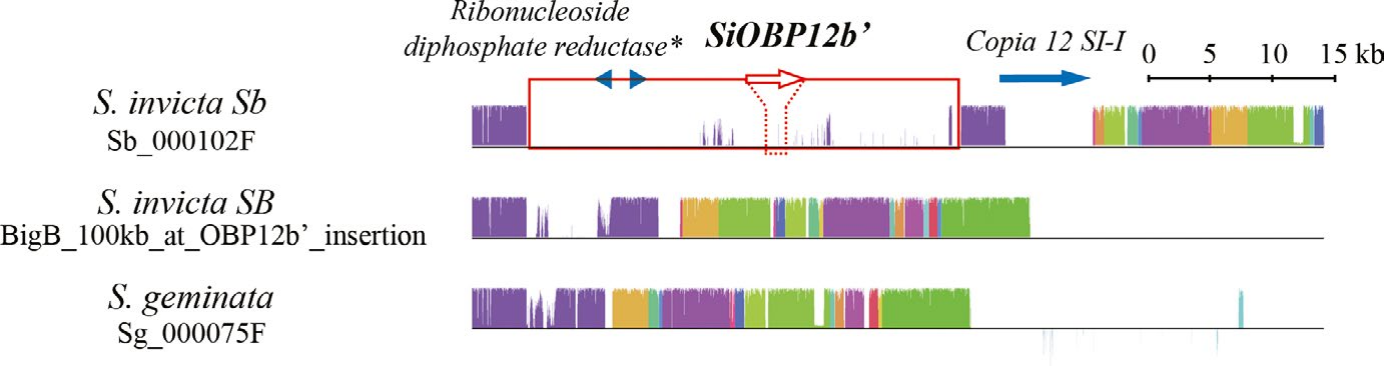
Mauve alignment of the *SiOBP12b*′ region in the genomes of *S. invicta Sb* and *SB* as well as *Solenopsis geminata*. The ~30‐kb region (empty red box) with * SiOBP12b*′ (empty red arrow) is absent in both the *SB* and *S. geminata* genomes, indicating that *SiOBP12b*′ likely inserted into the *Sb* genome after the split between the *S. invicta* and *S. geminata* lineages. This ~30‐kb region also contains two pseudogenized reductase genes (blue arrowheads); other predicted genes outside this region are not shown except for a *Copia* transposable element (blue arrow). Blocks with the same color in each genome indicate corresponding regions among the genomes. The height of each column in the blocks indicates percent similarity among the genomes. Blocks above or below the black lines indicate inverted regions compared to *S. invicta Sb*. Gaps between colored blocks indicate insertions/deletions or divergence. In the *Sb* genome, the insertion is on contig 000102F (Sb_000102F): positions 702901–736721 and corresponds to a manually assembled contig in the *SB* genome (BigB_100kb_at_OBP12b′_insertion; see Figure S8 for derivation of this contig): positions 59685–66456 and contig 000075F in the *S. geminata* genome (Sg_000075F): positions 1074960–1077317. *SiOBP12b*′ is located at 722120–725425 nt

In contrast to *SiOR463*, *SiOBP12* was expressed ~24‐fold higher in the antennae of *SB/Sb* compared to *SB/SB* workers. This higher expression was not from either of the *SB* or *Sb* alleles of *SiOBP12* (*SiOBP12B* and *SiOBP12b*, respectively) but rather from a duplicated (paralogous) copy of *SiOBP12*, which we call *SiOBP12b′* (corresponding to *SiOBPZ5* (Pracana et al., 2017)), based on four lines of evidence. First, all of the mapped reads from the *SB/Sb* samples at the *SiOBP12* locus contained mismatches, which corresponded to neither the *SB* (reference genome) nor *Sb* alleles (Table S3). Second, we conducted a rapid amplification of cDNA ends (RACE) assay on antennal expressed *SiOBP12* and found that the expressed transcript has an extension of ~170 bp of the 5’‐UTR compared to *SiOBP12B* (NCBI HQ853360). This extension mapped to a different genomic region (contig 000102F in the *Sb* PacBio genome). Third, copy number variation analysis indicated that there is twice the number of copies of *SiOBP12* in the *Sb* compared to the *SB* genome (Table S4). Finally, based on a new *Sb* PacBio assembly, we confirmed that this *SiOBP12* paralog is *Sb*‐specific and located within the supergene (Figure 3; Figure S8).

The *SiOBP12b′* gene could have been duplicated after the supergene was formed in an ancestor of *S. invicta*, and hence be specific for the *Sb* supergene allele, or it could have been lost from the ancestral *SB* supergene allele (Ross, Krieger, & Shoemaker, 2003). In the former case, the *SiOBP12b′* flanking region should be adjoined, while in the latter case, we may be able to find *SiOBP12b′* (or its remnants) in the genome of *Solenopsis geminata*, an outgroup species to the South American socially polymorphic clade of fire ants that includes *S. invicta* (Gotzek, Clarke, & Shoemaker, 2010; Martins, Fernando de Souza, & Bueno, 2014). In contrast to a previous study, which suggested that *OBP12* in *S. geminata* (*SgOBP12*) was partially degenerated (Pracana et al., 2017), we found that the 17 kb OBP gene cluster containing *Gp‐9* (*Gp‐9*, *OBP4*, *OBP13*, and *OBP12*) is conserved in the two species (identity >95%, blast results in Dryad). However, the *SiOBP12b*′ region is a complex duplication of ~30 kb that is absent in the *S. geminata* genome while its 5′ and 3′ adjacent regions are conserved and separated by only a ~2 kb unconserved fragment (Figure 3). This result suggests that *SiOBP12b*′ appeared in *S. invicta* or its ancestral lineage after divergence from the *S. geminata* lineage, and is compatible with its appearance after the formation of the *SB* and *Sb* supergene alleles.

### 3.3 Possible neofunctionalization of SiOBP12b′

The fate of a gene duplicate is typically loss of one of the copies but sometimes neofunctionalization. While the *SiOBP12b*′ sequence has an intact open reading frame, *SiOBP12B* and *SiOBP12b* appeared to have become nonfunctional independently because of an early stop codon caused by a C‐to‐T substitution at the 46^th^ nucleotide relative to the start codon in *SiOBP12B* or a 17‐base frame‐shifting insertion in *SiOBP12b* (Pracana et al., 2017). The nonfunctional *SiOBP12b* allele is consistent with no (this study) or very low expression of this allele (1 of 32 *OBP12* reads; Gotzek et al., 2011; Table S5). In contrast, the predicted nonfunctional status of *SiOBP12B* was surprising because it is expressed and also differed from the initially published intact *SiOBP12B* sequence (NCBI RefSeq HQ853360; Gotzek et al., 2011). Inspection of the original RNA‐Seq dataset revealed that the erroneous *SiOBP12B* assembly was due to the collapse of reads from both the *SiOBP12B* and *SiOBP12b*′ alleles (Table S5). Furthermore, all *SiOBP12B* sequences examined from two invasive populations (seven unrelated *SB* males from Georgia, USA [Wang et al., 2013] and 12 families from Taiwan [Qiu et al., 2018 and this study]) have the C‐to‐T substitution, indicating that the premature stop codon is likely fixed in the invasive range.

The loss of *SiOBP12B* and *SiOBP12b* may be compensated (i.e., replaced) by *SiOBP12b*′ in which case the protein sequence of the parental SiOBP12 and SiOBP12b′ should be highly similar. Alternatively, amino acid changes at functionally important residues may indicate neofunctionalization. Although both *SiOBP12B* and *SiOBP12b* have premature stop codons, their degeneration appears to be recent because both have high nucleotide and amino acid similarities to the ortholog *SgOBP12* and paralog *SiOBP12b*′. Thus, mutations in *SiOBP12b*′ can be inferred based on the patterns of substitutions in comparison among these sequences. We compared the four OBP12s to Gp‐9 (NCBI RefSeq Q8WP90) and the solved structure of the *B. mori* pheromone‐binding protein (NCBI RefSeq NP_001037494) and found 14 changes (8% of full‐length protein) in OBP12b′ relative to the presumptive ancestral sequence (Figure S9). Interestingly, six of these are located in alpha helix 5 including two at potentially important positions. The R117P change is potentially located in the binding pocket, and the R121H change is potentially at the pH‐sensitive conformation site. These amino acid differences may indicate that OBP12b’ has different ligand binding properties compared to OBP12B or OBP12b, rather than simply replacing the original function of OBP12.

Genes may also neofunctionalize in terms of gene expression. Prior studies have shown that many OBPs, including *Gp‐9* and *SiOBP12*, are expressed in nonantennal tissues (Morandin et al., 2016; Pracana et al., 2017; Zhang, Wanchoo, Ortiz‐Urquiza, Xia, & Keyhani, 2016). We tested whether *SiOBP12* (in an assay that cannot distinguish between paralogs) was also differentially expressed in the worker heads (without antennae), thoraces, and abdomens using qRT‐PCR assays. Similar to the well‐known *Gp‐9*, we found no expression differences between the three worker classes (Figure 2, all Tukey's HSD *p*‐values >0.05). Interestingly, the expression pattern of *SgOBP12* was similar to those of *SiOBP12B*—low expression in the antennae and at comparable levels relative to the internal reference genes in the heads, thoraces, and abdomens (Figure 2).

To determine whether the expression of *SiOBP12* in *SB/Sb* worker bodies is also primarily from *SiOBP12b*′ as in the worker antennae (which has 98% from *SiOBP12b*′ based on RNA‐Seq; Table S3), we inspected Sanger sequencing trace files of partial *SiOBP12* cDNA amplicons from the antennae, heads, and bodies (i.e., thorax–abdomen). We found a similar expression level of both *SiOBP12B* and *SiOBP12b*′ in the heads and bodies while, again, the antennae showed predominantly (or even pure) signal from *SiOBP12b*′ (Figure S10, all three bioreplicates in Dryad). Together, these results suggest that *SiOBP12b*′ may have acquired a new antennal regulatory element.

## 4 DISCUSSION

This study compared gene expression levels in the antennae of the fire ant workers of alternate social supergene genotypes and social forms in order to identify candidate genes that explain how *SB/SB* and *SB/Sb* workers differ in discriminating between *SB/SB* and *SB/Sb* queens. By targeting the most relevant sensory organ associated with this behavior, our study provided better sensitivity to detect tissue‐specific differences compared to similar studies which used the whole bodies of workers (Wang et al., 2008) and queens (Nipitwattanaphon, Wang, Dijkstra, & Keller, 2013). Other transcriptomics experiments have been conducted in fire ants in other contexts (Calkins et al., 2018; Chen, Shen, & Lee, 2006; Morandin et al., 2016; Nipitwattanaphon et al., 2014; Qiu et al., 2018). Our results and follow‐up experiments uncovered an interesting, differentially expressed OBP, *SiOB12b*′, whose expression patterns suggest that it may play a role in the differences between monogyne and polygyne colony forms in fire ants.

### 4.1 Antennal gene expression differences between the three worker classes are mainly from transposable elements, chemical metabolism, and perception genes

The antennae are the main sensory organ of insects, and thus, the genes expressed there have a large effect on what insects can detect and consequently how they behave (Athrey et al., 2017; Chen et al., 2017; McBride et al., 2014; Zhao et al., 2016). Our analysis identified 81 DEGs in the antennae across three worker classes (monogyne *SB/SB*, polygyne *SB/SB*, and polygyne *SB/Sb*). Globally, our results are consistent with previous studies of gene expression comparing different social supergene genotypes on whole bodies of queens, workers, and males, in that we also found an over‐representation of DEGs located in the supergene (Nipitwattanaphon et al., 2013; Wang et al., 2008, 2013). Despite supergene over‐representation, there was no overlap for the DEGs located within the supergene found by the antennal and worker whole‐body experiments. Thus, many other DEGs within the supergene likely remain to be found by supergene genotype comparisons of other tissues and developmental times. Another general pattern found in whole‐body worker gene expression profiles was that social environment had a stronger effect on gene expression than supergene genotype (Wang et al., 2008, 2013). However, we found that antennal expression was comparably affected by these two factors. A possible explanation for this difference is that by examining a specific organ (the antennae), we excluded many other organs which may be affected more by the social environment. For example, *Thelohania solenopsae* infection and gut symbiotic microbes tend to be found among polygyne colonies (Lee, Husseneder, & Hooper‐Bùi, 2008; Oi, Valles, & Pereira, 2004) while *Wolbachia* infections were higher in monogyne colonies (Shoemaker et al., 2003).

In fire ants, several TEs are presumably active based on their germline expression and many TEs have substantial expanded in the *Sb* haplotype (Lee & Wang, 2018; Stolle et al., 2019; Wang et al., 2013). Our study identified ten TEs that were differentially expressed based on genotype. This general expression pattern could be explained either by supergene allele‐specific gene regulation or by copy number variation of TEs between *SB* and *Sb* genomes. The general consensus is that most TE insertions are neutral or slightly deleterious; however, TEs that cause adaptive phenotypes have been described in many organisms, including insects (van't Hof et al. 2016; Jangam, Feschotte, Betrán, 2017; Li et al. 2018). Thus, it would be interesting to determine whether the differential expression of these TEs is just nonfunctional noise or adaptive gene regulation, especially for those associated with social form. Similarly, identifying the exact TE copy (or copies) that might contribute to the queen discrimination behavior would be an interesting avenue for future study.

Of the remaining DEGS, 13 encode plausible candidates that may explain how *SB/SB* and *SB/Sb* workers differ in discriminating between the *SB/SB* and *SB/Sb* queen chemical cues. The candidates include genes for both chemical metabolism (fatty acid synthase, cytochrome P450 enzymes, and esterase) and odorant perception (i.e., an OBP and an OR) (Table S2). Workers rely on the semiochemical cues displayed on the queen's cuticle to recognize a queen's genotype (Eliyahu et al., 2011; Keller & Ross, 1998; Trible & Ross, 2016). Therefore, the presence of DEGs that detect or metabolize chemicals was expected, due to their potential roles in a chemical sensing pathway, starting from chemical transport, continuing to receptor binding, and ending with chemical degradation. A genetic change at any step could affect insect behavior through a change in chemical sensitivity (transport and binding steps) or chemical modification (degradation), which in turn could affect receptor availability (Van den McBride et al., 2014; Montague, Mathew, & Carlson, 2011; Nakagawa, Sakurai, Nishioka, & Touhara, 2005; Ozaki et al., 2005; Pelletier et al., 2010; Berg & Ziegelberger, 1991). For example, OBPs (and CSPs) have roles in filtering and maximizing the signal through odorant solubilization (Leal, 2013; Van den Berg & Ziegelberger, 1991). Similarly, metabolism genes (e.g., esterases and cytochrome P450 enzymes) were found to degrade sex pheromones and volatile environmental chemicals possibly to reset receptor sensitivity in the antennae of moth and beetles, respectively (Cano‐Ramírez et al., 2013; Keeling et al., 2013; Vogt & Riddiford, 1981; Wojtasek & Leal, 1999). While it remains to be tested, we suggest that the chemical metabolism and odorant perception genes identified may function similarly to the examples above.

### 4.2 *SiOBP12b*': A candidate for queen discrimination?

Chemical binding is the very first step, in which the signal is filtered by an OBP (or CSP) before being transferred to an OR (Leal, 2013; Van den Berg & Ziegelberger, 1991). The OBP *Gp‐9* is a classic, although disputed, candidate that may have this function with regard to regulating social form differences (Gotzek & Ross, 2009; Gotzek, Shoemaker, & Ross, 2007; Keller & Ross, 1998; Krieger & Ross, 2002; Leal & Ishida, 2008). Our study showed that *Gp‐9* is not differentially expressed in the antennae or in different body parts between the three worker classes (Figure 3; see also Pracana et al., 2017). Previous studies showed that *Gp‐9* was highly expressed in polygyne queens compared to monogyne queens (Morandin et al., 2016; Pracana et al., 2017). Thus, differential expression of *Gp‐9* could possibly be important in queens but is unlikely in workers; functional differences in workers, if any, would have to be mediated by protein sequence differences.

**Figure 4:**
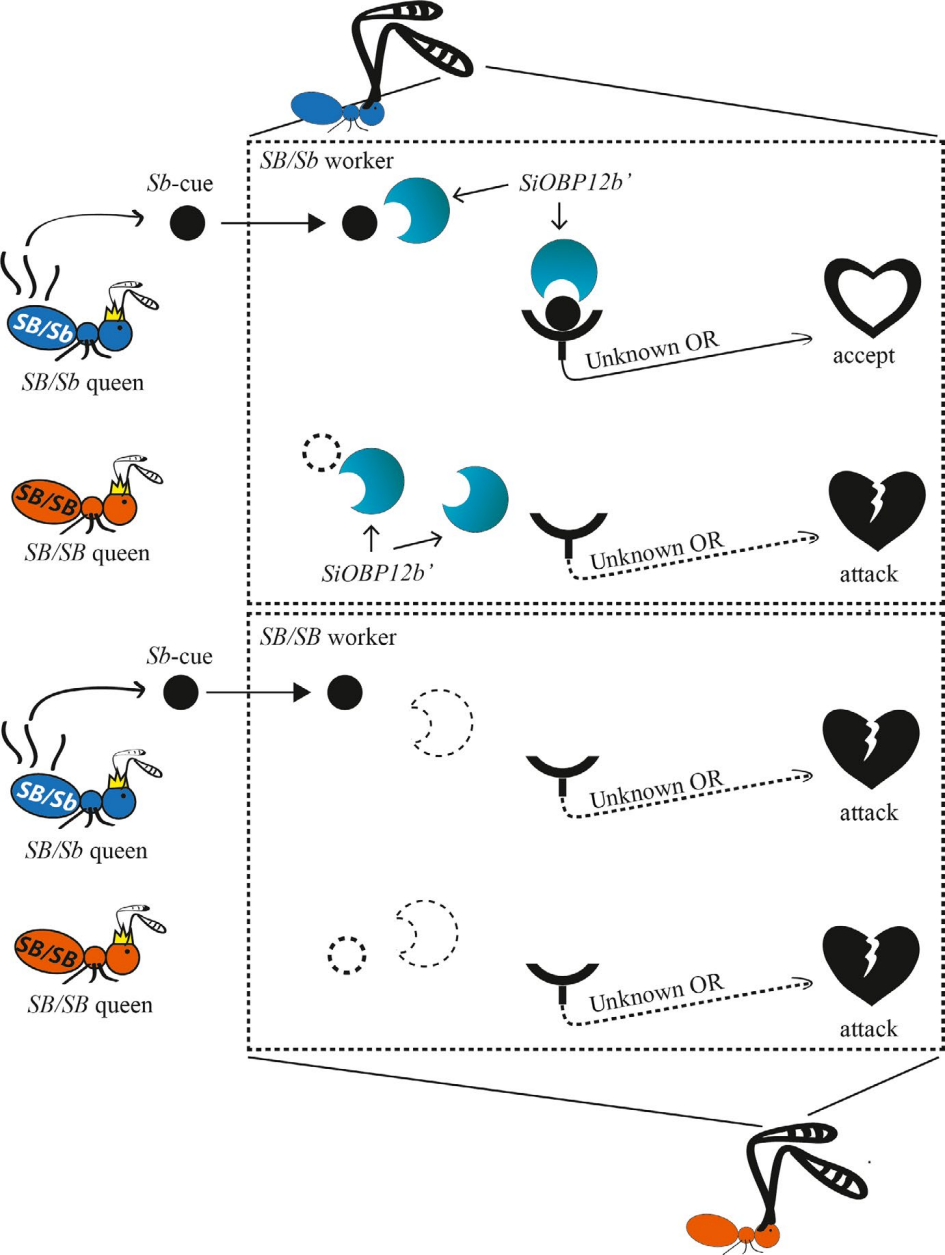
Model of how *SiOBP12b*′ functions in the queen discrimination behavior from the worker side. In this scenario, we propose that while *SB/Sb* queens gained an *Sb‐*specific queen chemical cue (black circle), *SB/Sb* workers evolved *Sb‐*specific *SiOBP12b*′ (blue half‐moon) which helps them to detect this cue. *SiOBP12b*′ in the *SB/Sb* worker antennae transfers the *Sb*‐specific queen chemical cue to an unknown odorant receptor (OR). The binding between the chemical cue–*OBP12b′* complex and OR signal acceptance (arrow pointing to the heart). In contrast, *SB/SB* queens who lack the *Sb*‐cue (dashed circle), cannot form the odorant‐binding complex, and, therefore, cannot transmit this signal (dashed arrow pointing to the broken heart). Similarly, the *SB/SB* workers, lacking *OBP12b′* (dashed half‐moon), cannot distinguish between *SB/SB* and *SB/Sb* queens

Although any of the 13 differentially expressed chemical metabolism and odorant perception genes has the potential to mediate the queen discrimination behavior, we suggest that *SiOBP12b′* may be the strongest candidate among these to explain the sensitivity of *SB/Sb* workers to introduced queens (Keller & Ross, 1998; Ross & Keller, 2002; Trible & Ross, 2016). Our results indicated that *SiOBP12b′*, an *Sb‐*specific gene, arose as a duplicate after the lineage leading to *S. invicta* split from that of *S. geminata*. Therefore, this gene could plausibly be involved in the social organization polymorphism in *S. invicta* and possibly the other socially polymorphic South American fire ants (Krieger & Ross, 2005). Based on the distinct and high expression in the antennae of *SB/Sb* individuals, we speculate that *SiOBP12b′* may provide a simple mechanistic explanation for the queen discrimination behavior (Figure 4). In our model, during the evolution of the supergene in the fire ant, the *SB/Sb* queens gained an “*Sb*‐cue” (e.g., possibly unsaturated hydrocarbons^, ^polar biomolecules^, ^or Gp‐9 (Eliyahu et al., 2011; Trible & Ross, 2016)) while the *SB/Sb* workers gained an “*Sb*‐cue” detector: *SiOBP12b*′. Binding between the “*Sb*‐cue” and SiOBP12b′ would form a complex, which would subsequently activate an unknown OR to transfer the signal of “Acceptance.” The *SB/SB* queens without the “*Sb‐*cue” fail to activate this “Acceptance” signal. In a polygyne colony, *SB/SB* workers, which do not have *SiOBP12b*′, respond by either ignoring introduced queens or following the “decision” of *SB/Sb* workers (Gotzek & Ross, 2008; Keller & Ross, 1998). In a monogyne colony, *SB/SB* workers accept only the dominant queen, which is their own queen. Future functional genetic experiments will be needed to test this model.

### 4.3 *SiOBP12b*′: Neofunctionalization, adaptation, or demography?

Our analysis also revealed that *SiOBP12b′* has gained a new antennal regulatory element. While expression in the head, thorax, and abdomen for *OBP12* occurs in both *S. geminata* and *S. invicta*, expression in the antenna occurs only in *S. invicta* workers, with *SiOBP12b*′ being the predominantly expressed paralog. A previous study indicated that *SiOBP12b*′ has experienced positive selection (Pracana et al., 2017). Examination of the protein sequence also revealed multiple amino acid site differences, including two affecting potential functional sites, which may affect OBP12b*′* ligand binding efficiency, regulation, and/or target identity. These changes may indicate neofunctionalization of a duplicated gene. Alternatively, this pattern might reflect a bottleneck in forming the invasive population coupled with adaptation. Further investigation of this gene in related species and functional confirmation of the amino acid changes between the ancestral OBP12 and SiOBP12b′ will help reveal the evolutionary history of these paralogs.

The contribution of neofunctionalized after gene duplication in the evolutionary divergence between non‐recombining supergene alleles is poorly understood. Supergenes and their evolution have many parallels with sex chromosomes (Graves, 2006; Liu, 2018). Neofunctionalization has been appreciated in the sex chromosome evolution literature. For example, many new sex chromosomes have evolved by the acquisition of a duplicate copy and subsequent neofunctionalization of the “trigger” genes in terrestrial vertebrates and fish (Matsuda et al., 2002; Smith et al., 2009). Additionally, several examples in both vertebrate and invertebrate sex chromosomes have demonstrated duplication and then neofunctionalization of genes for other processes on the Y (Ellegren & Parsch, 2007; Lahn, Pearson, & Jegalian, 2001; Liu, 2018). If confirmed, *SiOBP12b′* would represent one of the rare examples for nonsex chromosomes.

Does *SiOBP12b′* also have a function in the rest of the body? We found expression of *SiOBP12b′* in tissues outside of the antennae of the *SB/Sb* workers, and its expression in whole bodies of queens in other studies presumably also reflects at least some nonantennal gene expression (Morandin et al., 2016; Pracana et al., 2017). This adds to the growing list of OBPs and CSPs found to be expressed in nonantennal tissues (Calvello et al., 2003; Guo et al., 2011; Jacquin‐Joly, Vogt, François, & Nagnan‐Le, 2001; Ozaki, Morisaki, Idei, Ozaki, & Tokunaga, 1995). The potential functions of OBPs/CSPs in nonantennal body parts are still poorly understood, although there are some data. For example, OBPs have been found associated with leg regeneration in the American cockroach (Kitabayashi, Arai, Kubo, & Natori, 1998; Nomura, Kawasaki, Kubo, & Natori, 1992), visual pigment transportation in Lepidoptera (Zhu et al., 2016), and larval molting in *S. invicta* (Cheng, Lu, Zeng, Liang, & He, 2015). Our comparative expression results suggest that *SiOBP12b*′ has at least two functions: chemical perception in the antennae and an unknown, possibly ancestral, function in other tissues (e.g., replacement of the degenerated parental *SiOBP12*). Additionally, because *SiOBP12b*′ is only present in *SB/Sb* individuals, its nonantennal functional could also affect phenotypic differences between monogyne and polygyne colonies. The precise roles of this gene remain to be investigated.

## 5 CONCLUSIONS

In summary, we have profiled gene expression of the fire ant worker antennae and found that supergene genotype and social environment equally affected antennal gene expression. We identified 81 DEGs including 13 putative metabolism and odorant perception genes that may be involved in queen discrimination. Of these, we found that *SiOBP12b*′ is a particularly interesting candidate because it is an *Sb*‐specific paralog that has acquired high expression in the antennae. These genes, and especially *SiOBP12b*′, will be the subject of further behavioral genetic analyses.

## CONFLICT OF INTEREST

None declared.

## AUTHORS’ CONTRIBUTIONS

V.D.D. and J.W. conceived and designed the experiments. V.D.D. conducted experiments. All authors contributed to bioinformatic analyses. V.D.D. and J.W. wrote the manuscript. All authors gave final approval for publication.

## Data Availability

The GenBank accession for the raw and processed RNA sequence data is http://www.ncbi.nlm.nih.gov/geo/query/acc.cgi?acc=GSE126684 and that for the *SiOBP12b′* cDNA sequence is MN193778. The accession numbers of the fire ant PacBio genomes are SAMN11869237 (*SB*) and SAMN11869238 (*Sb*). Additional auxiliary data are deposited in Dryad at https://doi.org/10.5061/dryad.qn4d68k.
