# Supplementary material for: Has gene expression neofunctionalization in the fire ant antennae contributed to queen discrimination behavior?
Source: Ecol Evol. 2019 Oct 29;9(22):12754–66. doi: 10.1002/ece3.5748 (PMC6875580; doi:10.1002/ece3.5748)
Supplement: Supplementary file 1 [file ECE3-9-12754-s001.pdf]

Figure S1

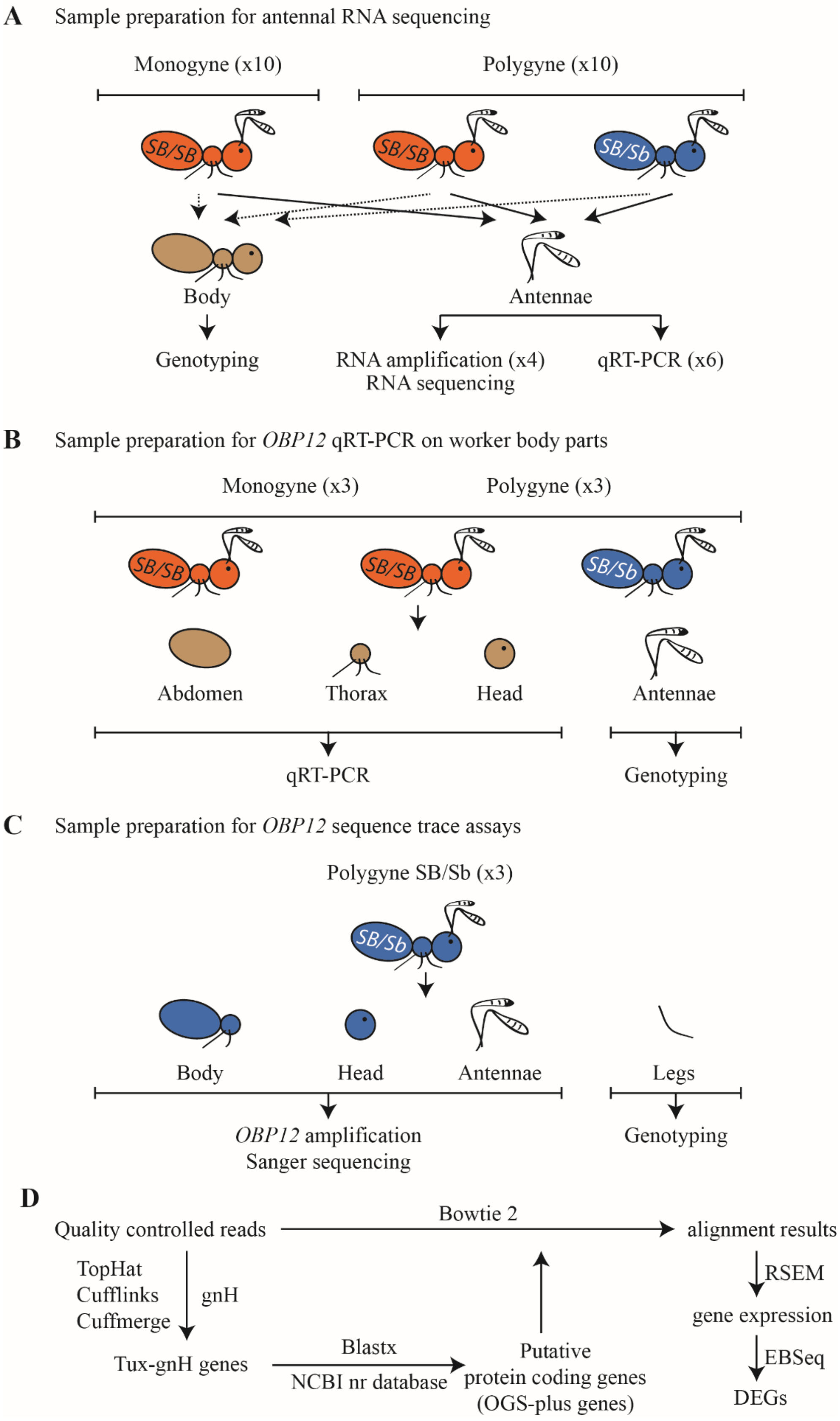
A
Sample preparation for antennal RNA sequencing
Monogyne (x10)
Polygyne (x10)
SB/SB
SB/SB
SB/Sb
Body
Antennae
Genotyping
RNA amplification (x4)
RNA sequencing
qRT-PCR (x6)
B
Sample preparation for OBP12 qRT-PCR on worker body parts
Monogyne (x3)
Polygyne (x3)
SB/SB
SB/SB
SB/Sb
Abdomen
Thorax
Head
Antennae
qRT-PCR
Genotyping
C
Sample preparation for OBP12 sequence trace assays
Polygyne SB/Sb (x3)
SB/Sb
Body
Head
Antennae
Legs
OBP12 amplification
Sanger sequencing
Genotyping
D
Quality controlled reads
Bowtie 2
alignment results
TopHat
Cufflinks
Cuffmerge
gnH
RSEM
gene expression
EBSeq
DEGs
Tux-gnH genes
Blastx
NCBI nr database
Putative
protein coding genes
(OGS-plus genes)

Figure S1. Preparation of different body parts for RNA extraction and RNA-seq analysis pathway. **(A-C)** To separate the different genotypes of *S. invicta*, a genotyping step is required. **(A)** To prepare antennal RNA, workers were genotyped individually using the body. In each bio-replicate, antennae of the same worker class were pooled to extract RNA. Ten bio-replicates of antennal RNA were used in RNA amplification and sequencing (x4, pool of > 39 antennal pairs) and qRT-PCR assays (x6, pool of 20 antennal pairs). **(B)** To prepare RNA of different body parts for qRT-PCR, we genotyped individually using the antennae. In each bio-replicate, we extracted RNA from pools of 15 heads (thoraces or abdomens) of the same worker class. **(C)** Preparation of RNA from different body parts for *SiOBP12* sequence trace assays. Polygyne workers were genotyped individually using two random legs. We extracted RNA from the antennae, heads, and bodies (i.e., thorax-abdomen) of *SB/Sb* workers (20 individuals). Three bio-replicates were conducted in both experiments (B) and (C). The four body parts (antennae, heads, thoraces, and abdomens) of *S. geminata* were prepared together without a genotyping step. **(D)** Antennal RNA-seq analysis pathway. Quality controlled reads were first mapped onto the fire ant genome gnH following the Tuxedo pipeline to generate the Tux-gnH genes. We compared Tux-gnH genes with the NCBI non-redundant database using blastx and retained putative coding genes (OGS-plus genes). The quality controlled reads were mapped onto OGS-plus genes using Bowtie 2. Gene expression level was estimated based on this mapping using RSEM. Differentially expressed genes were tested by the EBSeq package.

Figure S2

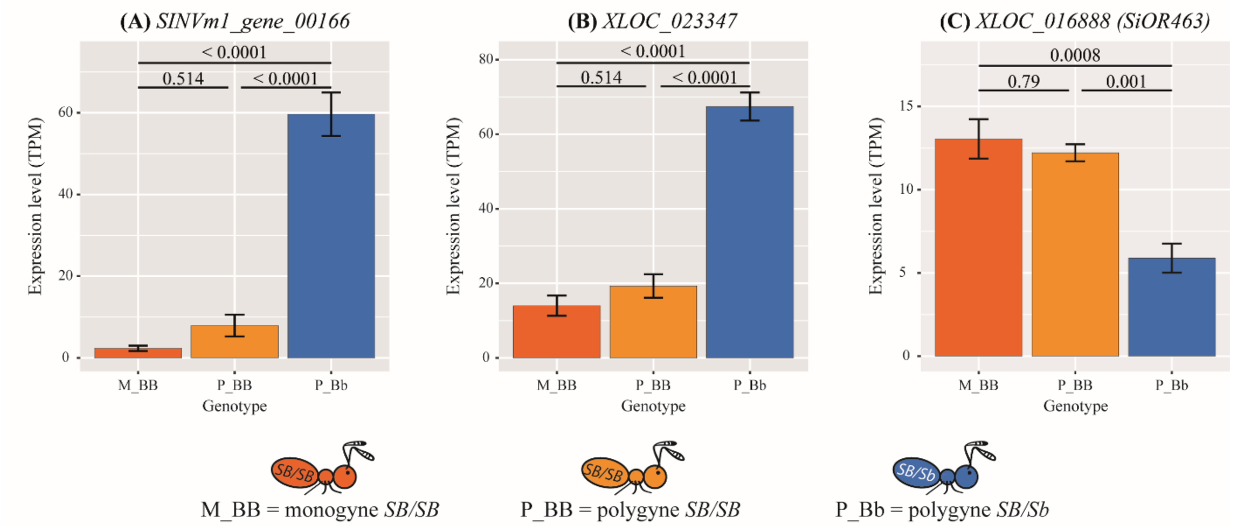

Figure S2. Expression of *SINVm1_gene_00166*, *XLOC_023347*, and *XLOC_016888* (*SiOR463*) in the worker antennae of three worker classes. Barplot values are TPM (Transcripts Per Million) as estimated by RSEM. *P*-values for each pairwise comparison were determined using the post-hoc Tukey HSD's test. **(A & B)** Two genes, *SINVm1_gene_00166* and *SINVm1_gene_023347*, were regulated primarily by genotype although statistical tests of differential expression by EBSeq suggested that these genes were expressed differently in all three pairwise comparisons of the three worker classes (probability of the pattern > 94%, posterior probability of being differentially expressed > 99%). This discordance may due to the small sample size of the study. **(C)** The expression level of *SiOR463* in *SB/Sb* individuals compared to *SB/SB* individuals is approximately half. Its differential gene expression may simply reflect gene dose as SiOR463 has been deleted in the Sb genome.

Figure S3

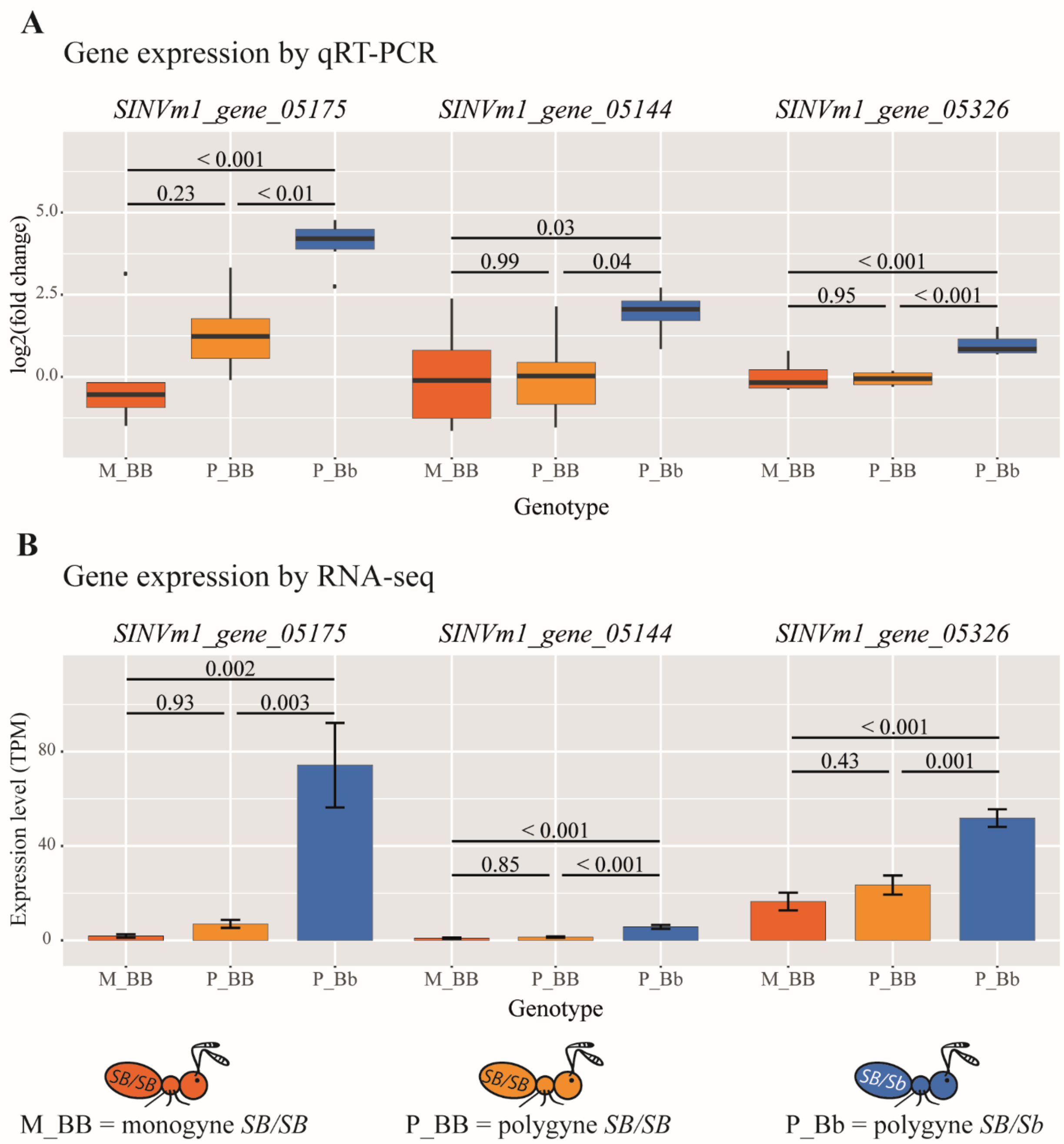

Figure S3. Gene expression validation by qRT-PCR in the worker antennae on three of the seven DEGs located in the supergene for the three worker classes: monogyne *SB/SB*, polygyne *SB/SB* and polygyne *SB/Sb*. The putative functions of *SINVm1_gene_05175* and *SINVm1_gene_05326* are nadh dehydrogenase and coiled-coil domain containing protein, respectively. The function of gene *SINVm1_gene_05144* is unknown. Four bio-replicates were conducted with each containing 24 to 52 pairs of antennae. Consistent with RNA-seq analysis,

qRT-PCR showed that all three genes were up-regulated in *SB/Sb* individuals compared to the other two worker classes. **(A)** Relative gene expression levels by qRT-PCR shown as $\Delta\Delta$Ct ($\log_2$ fold change), *P*-values are from post-hoc Tukey's tests. **(B)** Gene expression values from RNA-seq in TPM (Transcripts Per Million) as estimated by RSEM. Testing by EBSeq showed these genes were highly expressed in P_Bb compared to both M_BB and P_BB (probability of the pattern > 90%, posterior probability of being differentially expressed > 99%). Pairwise comparisons used the post-hoc Tukey's test.

Figure S4

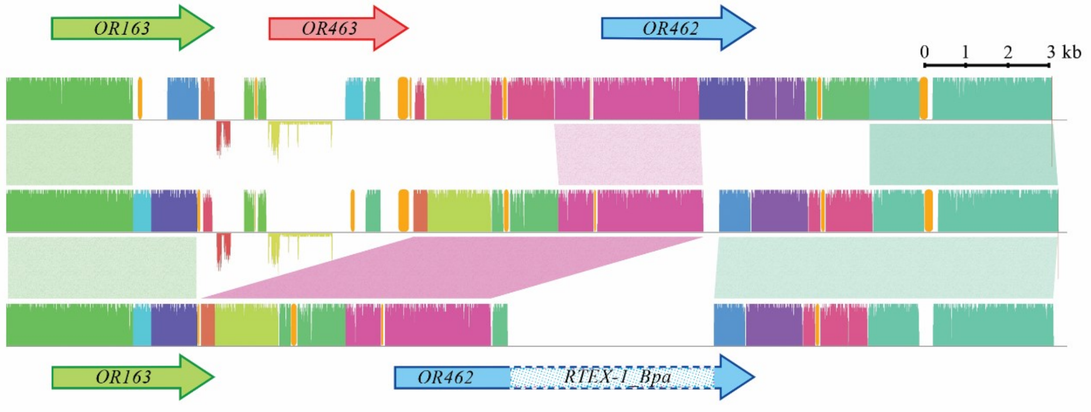

Figure S4. Mauve alignment of the OR cluster (*OR163*, *OR463,* and *OR462*) in the genomes of *S. geminata* as well as *S. invicta SB* and *Sb*. Both *OR163* (green arrow) and *OR462* (blue arrow) are found in the three genomes while *OR463* (red arrow) is absent in the *Sb* genome, indicating that the region was deleted probably after the formation of the supergene. *SiOR462b* has a ~5 kb insertion in the sixth exon, which is a putative RTEX-1_Bpa retrotransposon (see also figure S7). Blocks with the same color in each genome indicate corresponding regions among the genomes. The height of each column in the blocks indicates percent similarity among the genomes. Ribbons show the location of blocks in different genomes. Blocks above or below the black lines indicate inverted regions compared to *S. invicta Sb*. Gaps between colored blocks indicate insertions/deletions or divergence.

Figures S5-S7. IGV plots of low coverage genomic sequence reads from seven pairs of males (*SB* and *Sb* brothers) mapped onto the OR cluster region containing *SiOR163*, *SiOR463,* and *SiOR462*. The three ORs depicted are *SiOR163* (green arrow), *SiOR463* (red arrow) and *SiOR462* (blue arrow). Each panel (row) is the mapping results of one male genome with the read coverage of each base (upper track) and the read alignment (lower track) information. The coverage and SNPs are shown by the height and the colors of bars in the coverage track, respectively. In the alignment track, reads are colored by insertion size and pair orientation where red reads indicate deletion and blue reads indicate inverted duplication. Because the whole genome was aligned onto only one PacBio genome contig, repetitive sequences have higher coverage and therefore show SNPs compared to the adjacent regions.

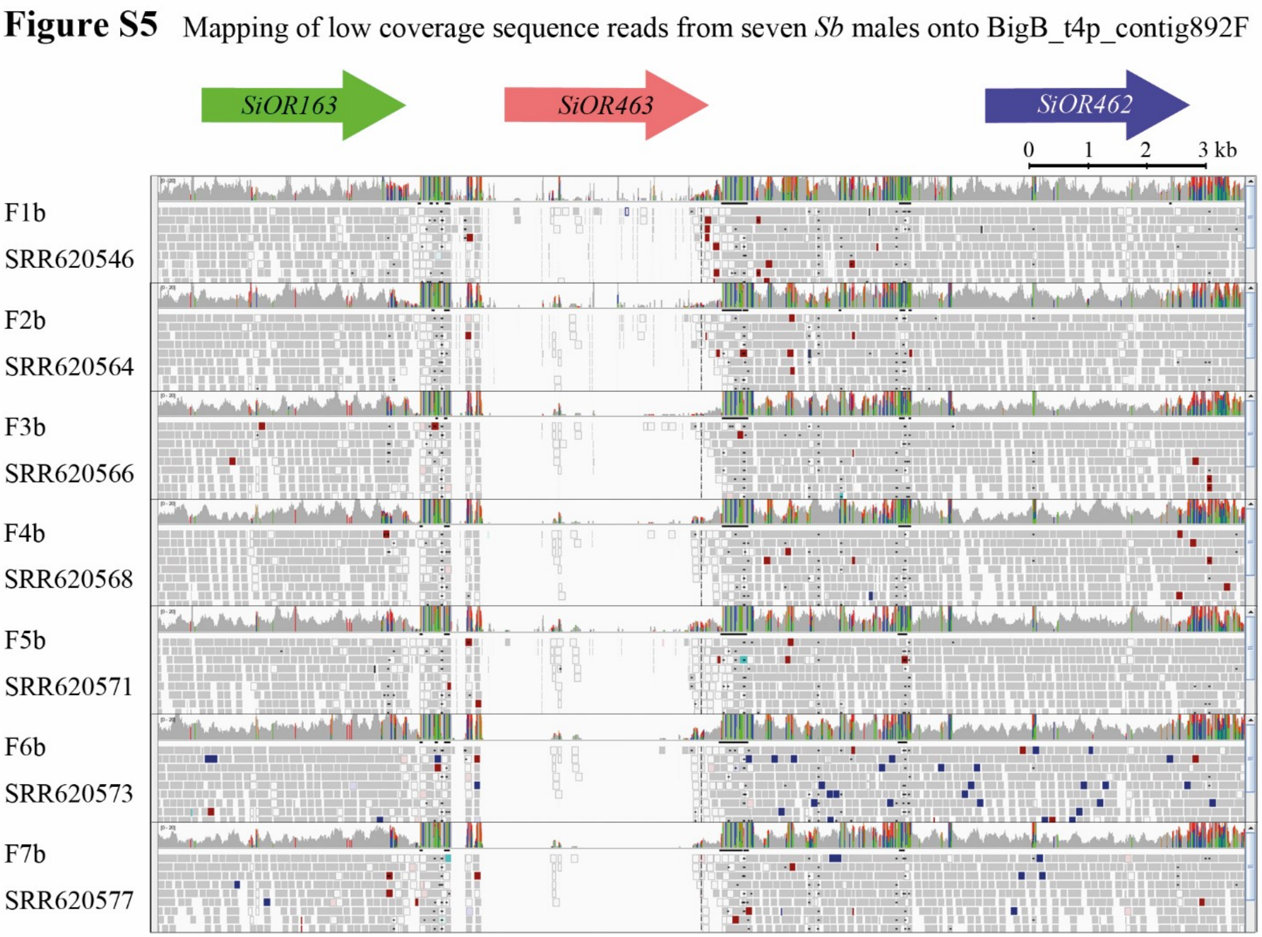

Figure S5. Mapping of low coverage sequence reads from seven *Sb* males onto the *SB* PacBio assembly contig 892F. The few discrete reads mapping to the *SiOR463* locus are likely repeat sequences.

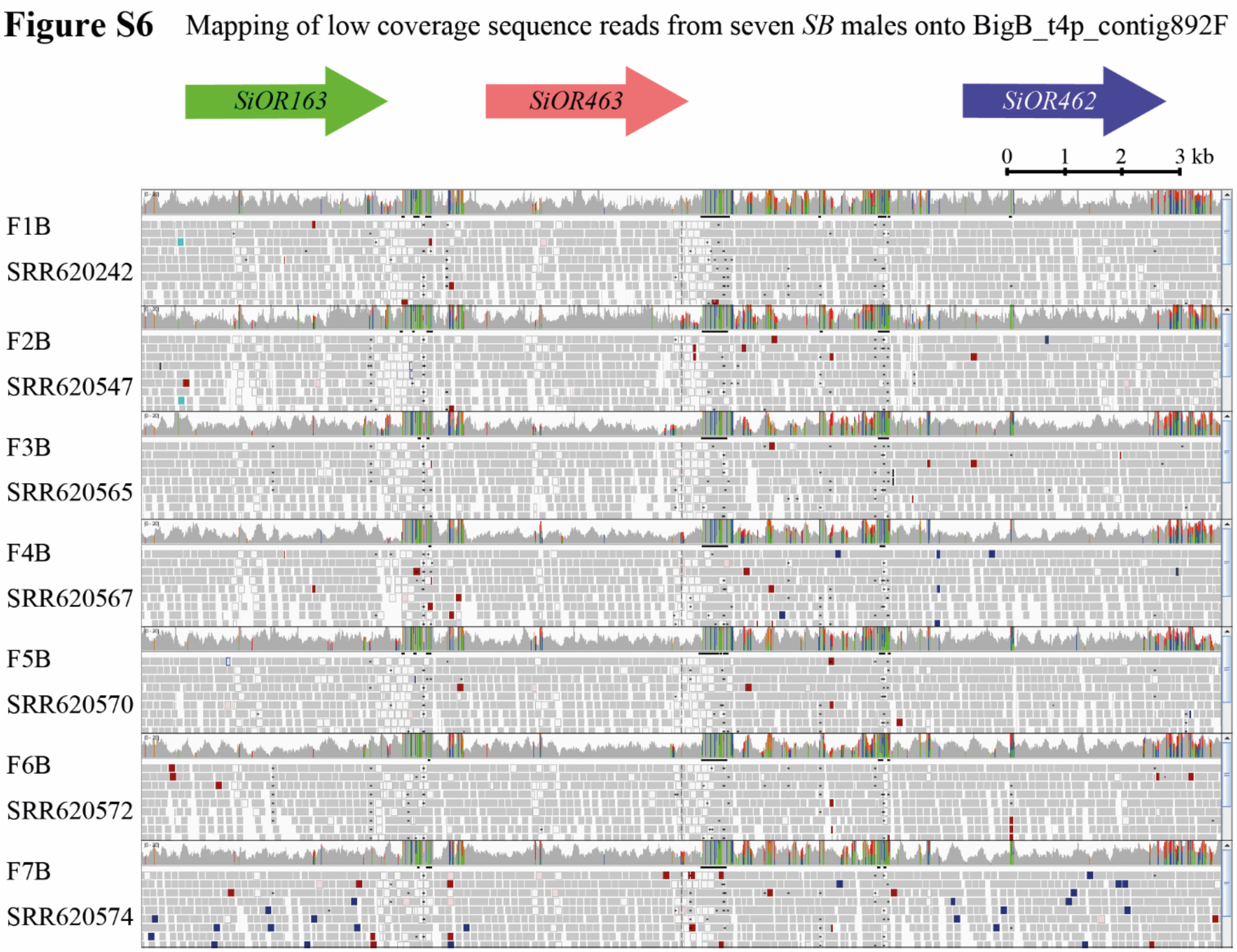

Figure S6. Mapping of low coverage sequence reads from seven *SB* males onto the *SB* PacBio assembly contig 892F.

Figure S7 Mapping of low coverage sequence reads from seven *Sb* males onto littleb_t2p_contig200F

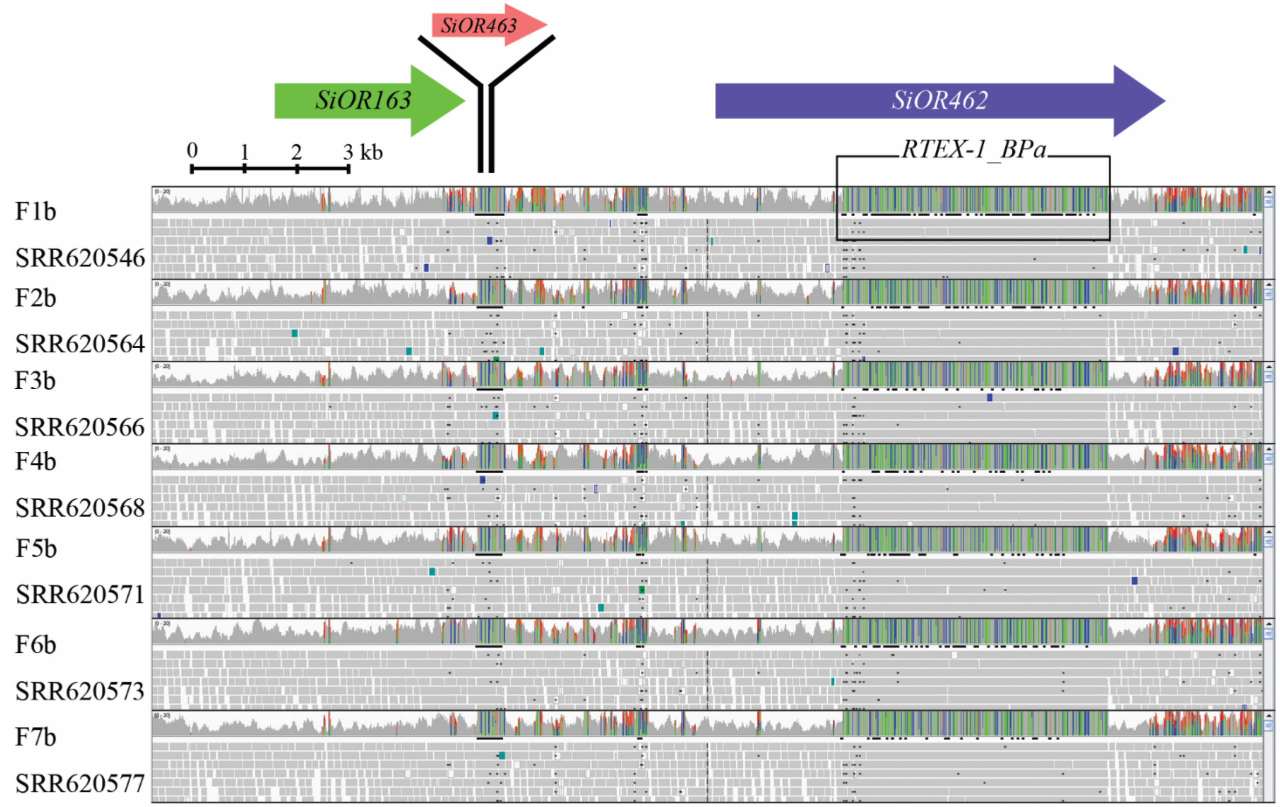

Figure S7. Mapping of low coverage sequence reads from seven *Sb* males onto the *Sb* PacBio assembly contig 200F. *SiOR462* has a ~5 kb insertion in the sixth exon, corresponding to a *RTEX-1_BPa* retrotransposon (boxed, similarity > 99%). The insertion is predicted to produce a gene-retrotransposon chimera, associated with a frame shift and a premature termination codon. The deleted *SiOR463* is indicated with the red arrow and the funnel (not to scale).

Figure S8

Step 1: Determined scaffold and contigs on the gnH and *SB* PacBio genome assemblies that corresponded to the *Sb* contig containing OBP12b'

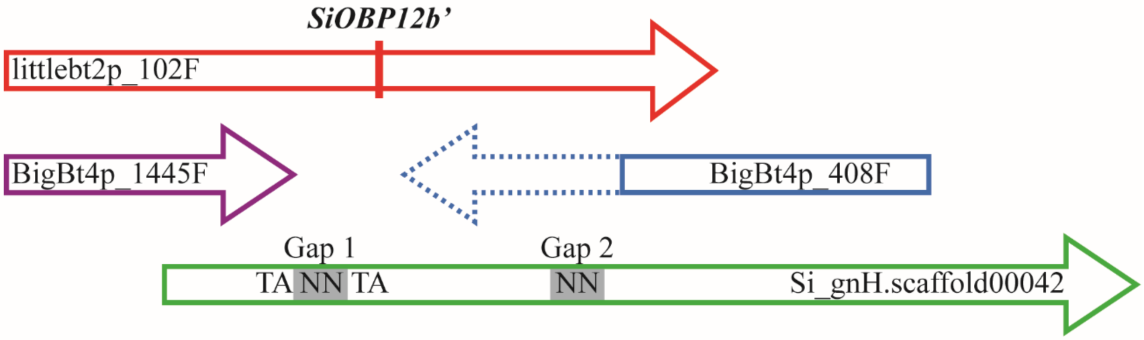

Step 2: Clipped off one end of BigBt4p_408F due to an assembly error

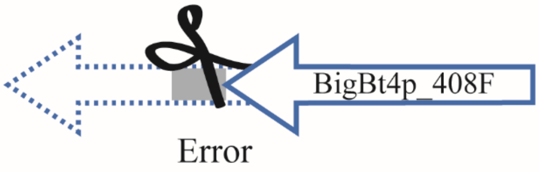

Step 3: Mapped SB PacBio genome raw reads onto Si_gnH.scaffold00042 Identified PacBio reads that cross Gap1 and Gap2

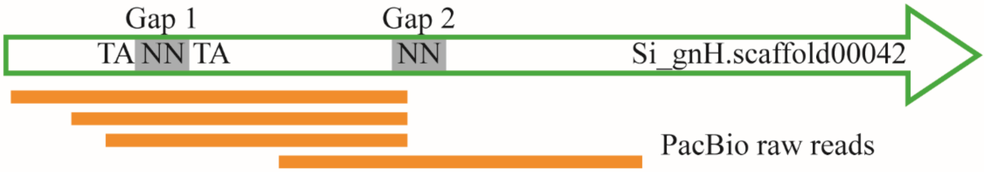

Step 4: Corrected sequence using Si_gnH.scaffold00042 and Si_gnH.contig28264

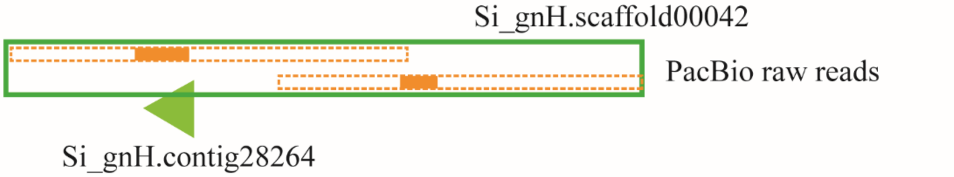

Step 5: Scaffolded BigBt4p_1445F and BigBt4p_408F using corrected Si_gnH.scaffold00042

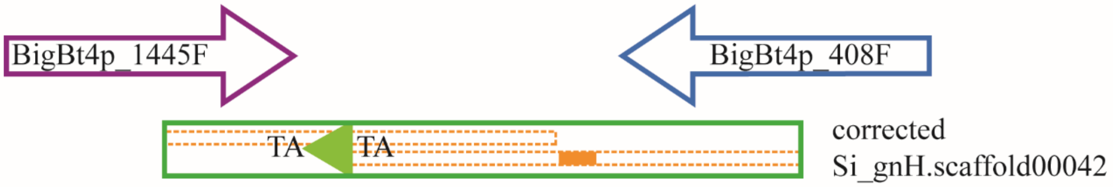

Step 6: Merged and truncated sequence to 100 kb

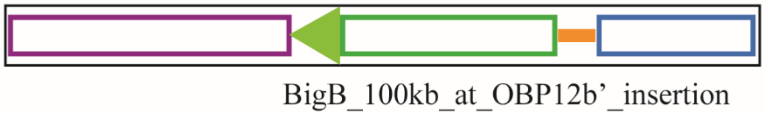

Figure S8. Schematic of the manual assembly process for the BigB_100kb_at_OBP12b'_insertion sequence. **Step 1**: *SiOBP12b'* (vertical red bar) is located on contig 102F (littlebt2p_102F, red arrow) in the Sb PacBio genome assembly version t2p. To determine the location of this region in the *SB* genome, we aligned littlebt2p_102F onto both the *SB* PacBio genome version t4p (BigBt4p) and Si_gnH assemblies. The *SiOBP12b'* region is located in a gap between contigs 1445F (BigBt4p_1445F, purple arrow) and 408F (BigBt4p_408F, blue arrow) of the BigBt4p genome; however, it is covered by scaffold00042 in the Si_gnH genome (Si_gnH.scaffold00042, green arrow), which itself has two gaps: Gap 1 and Gap 2 (grey blocks with NN). Gap 1 is surrounded by TA microsatellite repeats (TA), while Gap 2 seems to be a unique sequence. **Step 2**: We noticed that the 3'-end of the BigBt4p_408F contig does not match Si_gnH.scaffold00042 (dashed blue BigBt4p_408F). Detailed investigation into the region revealed an assembly error at a repeat (grey block), therefore we clipped off the 3'-end of BigBt4p_408F at the error site. **Step 3**: To determine the sequence of Gap 1 and Gap 2, we mapped the raw reads from *SB* PacBio whole genome sequencing onto Si_gnH.scaffold00042, and found four reads that cross Gap 1 (three reads) and Gap 2 (one read) (orange lines, read IDs in table S6). **Step 4**: Since the error rate of PacBio sequence is about 15% (Korlach 2015; Rhoads and Au 2015), we kept the sequence of Si_gnH.scaffold00042 and only filled the gaps with the PacBio read sequences (orange blocks: used sequences; dashed orange blocks: discarded sequences). We examined if the PacBio reads corresponded to any Si_gnH scaffolds. We found that contig28264 (Si_gnH.contig28264, green arrow) corresponded to Gap 1, and thus we filled Gap 1 with Si_gnH.contig28264. **Step 5**: We subsequently scaffolded BigBt4p_1445F and BigB_408F using the post-PacBio gap-filled Si_gnH.scaffold00042. **Step 6**: We truncated the sequence to a 100 kb contig, named BigB_100kb_at_OBP12b'_insertion. This contig contains the full length of BigBt4p_1445F (purple box), Si_gnH.contig28264 (green triangle), part of Si_gnH.scaffold00042 (green box), part of PacBio raw read (orange line) and part of BigBt4p_408F (blue box). The direction of the arrows and triangle show the direction from the 5'- end to 3'- end of the indicated sequences. Sketch is not to scale.

Figure S9

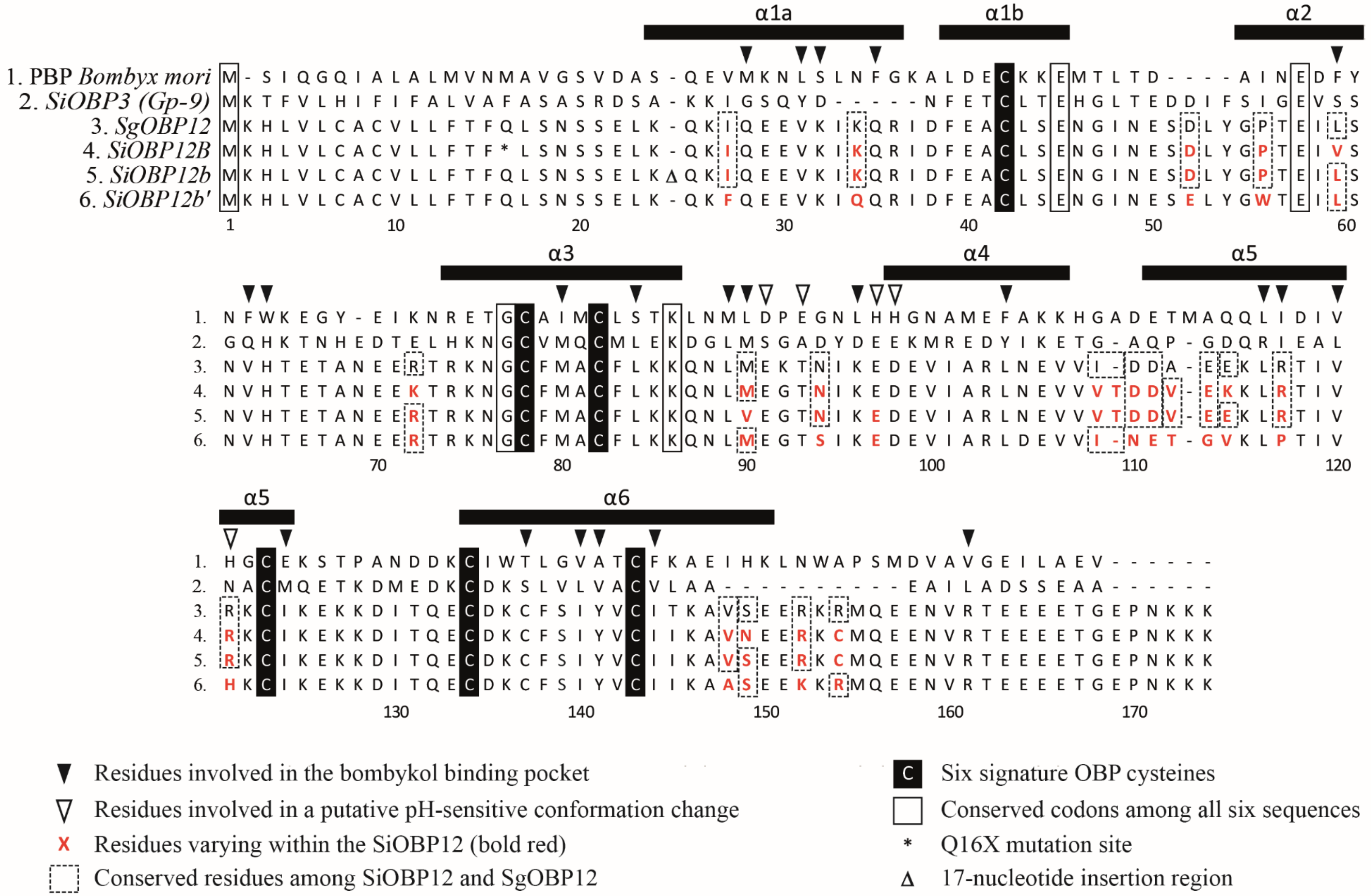

α1a
α1b
α2
α3
α4
α5
α6
1. PBP Bombyx mori
2. SiOBP3 (Gp-9)
3. SgOBP12
4. SiOBP12B
5. SiOBP12b
6. SiOBP12b'
Residues involved in the bombykol binding pocket
Residues involved in a putative pH-sensitive conformation change
Residues varying within the SiOBP12 (bold red)
Conserved residues among SiOBP12 and SgOBP12
Six signature OBP cysteines
Conserved codons among all six sequences
Q16X mutation site
17-nucleotide insertion region

Figure S9. Multiple sequence alignment of OBP12 proteins in *S. invicta* and *S. geminata*, SiGp-9, and the *Bombyx mori* Pheromone Binding Protein (PBP). Residues putatively involved in the binding site and pH-sensitive conformation change in the protein structure, based on the *B. mori* PBP, are indicated by black and empty arrow heads, respectively. Fourteen amino acid changes are specific to SiOBP12b', however, only R117P and R121H (coordinates relative to the 1st codon of SiOBP12b') are predicted to have a potential functional affect. Protein sequence: *B. mori* PBP (NP_001037494), SiGp-9 (Q8WP90), SiOBP12B (gnH, Wurm et al. 2011), SiOBP12b (*Sb* genome, Wang et al. 2013), SiOBP12b' (RACE assays in this study, NCBI RefSeq MN193778), and SgemOBP12 (*S. geminata* genome, Dryad). Amino acid variation among SiOBP12 (B, b, b') protein sequences are in bold red. Conserved amino acids among all six sequences are in solid boxes, with the six signature OBP cysteines also highlighted with black background. The conserved amino acids among SiOBP12 (B, b, and b') and SgOBP12 are in dashed boxes.

Figure S10

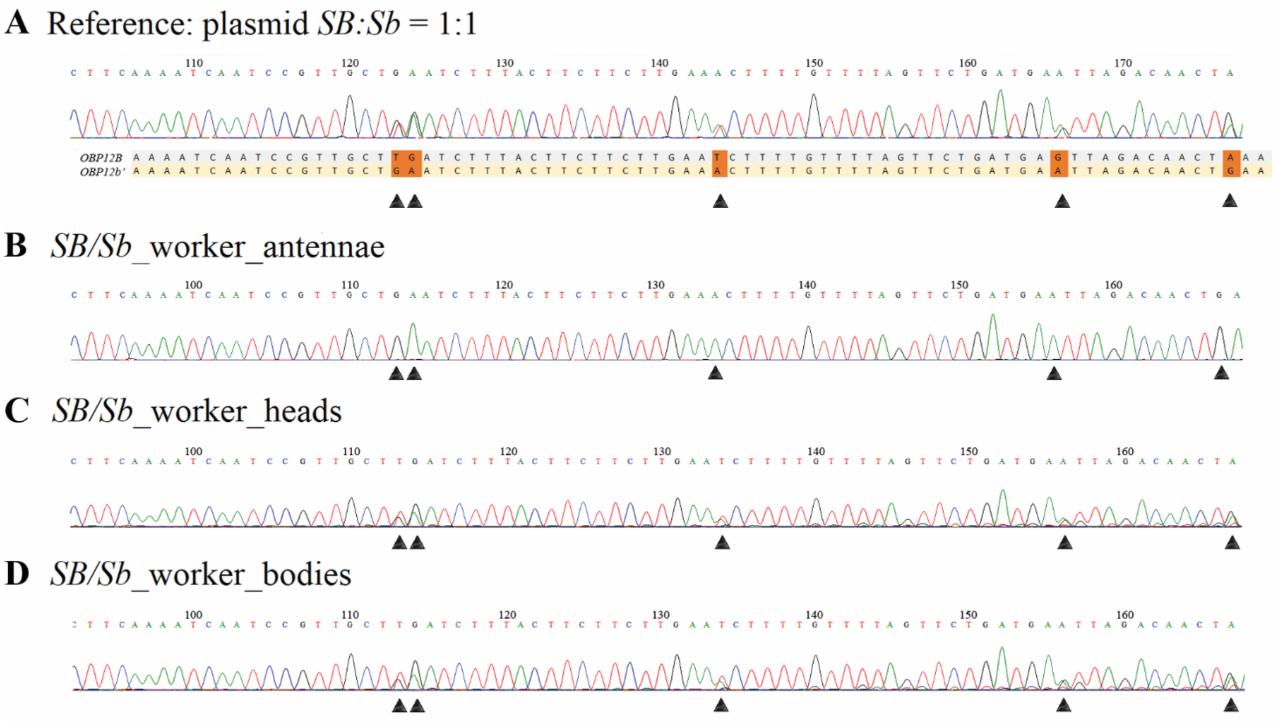

Figure S10. Example of the sequence traces showing the expression of *SiOBP12* in the worker antennae, heads, and bodies. Common fragments of *SiOBP12B* and *SiOBP12b'* were amplified using shared PCR primers. A pure sequence signal indicates the expression of one of the two genes while a mixed signal with similar peak heights indicates a similar expression level. *SiOBP12B* and *SiOBP12b'* specific SNP sites can be seen (black arrow heads). This assay showed that worker antennae expressed predominantly *SiOBP12b'* while worker heads and bodies expressed both *SiOBP12B* and *SiOBP12b'* at similar levels. (A) A mixture of *SiOBP12B* and *SiOBP12b'* cDNA plasmids at a 1:1 ratio was used as an artificial control. The trace is to show that a mixed signal of *SiOBP12B* and *SiOBP12b'* can be seen if they were expressed at a similar level. (B) *SiOBP12* sequence trace assay

on the worker antennal RNAs showed a pure signal of *SiOBP12b'*. (C & D) *SiOBP12* sequence trace assay on the worker head and body RNA showed mixed signals of *SiOBP12B* and *SiOBP12b'*, similar to the control. We extracted RNA from antennae, heads, and bodies of the same pool of *SB/Sb* individuals from the same polygyne colony. Three bio-replicates (colonies) were conducted (for all the sequencing results, see Dryad).
