# Supplementary material for: Has gene expression neofunctionalization in the fire ant antennae contributed to queen discrimination behavior?
Source: Ecol Evol. 2019 Oct 29;9(22):12754–66. doi: 10.1002/ece3.5748 (PMC6875580; doi:10.1002/ece3.5748)
Supplement: Supplementary file 2 [file ECE3-9-12754-s002.pdf]

Table S1. Antennal RNA-seq library summary

Each library is a pool of 39.5 to 53 pairs of antennae of the same *Gp-9* genotype and from the same colony

[a]Number of read pairs produced by Illumina sequencing using the HiSeq platform

[b]Number of read pairs after quality control by Cutadapt (-q 10 -m 30)

[c]Number of read pairs mapped onto the OGS-plus reference by Bowtie2

[d]Percentage of duplicate reads as assessed by mapping onto Si_gnH and samtools markdup

Sample_ID	Colony_ID	Library_ID	Antennal pairs	Social form	Genotype	Raw_reads[a]	Reads_pass_QC[b]	Mapped_reads[c]	% Dup[d]
MA2	Rui7	LGC16_JH03A	39.5	Monogyne	*SB/SB*	29,197,782	25,511,428	4,217,789	31.6
MA3	Zhuang3	LGC_JH06	53			29,815,211	29,165,789	4,691,160	14.1
MA6	Tan1	LGC_JH09	40			30,629,100	29,932,588	6,137,927	16.6
MA7	Rui1	LGC16_JH12	40.5			23,908,640	22,795,417	2,535,042	16.5
PAM2	Rui9	LGC_JH02	39.5	Polygyne		27,570,550	26,707,126	4,337,375	27.2
PAM3	Gui3	LGC_JH05	53			29,718,335	28,817,724	4,786,740	13.6
PAM6	Bang3	LGC_JH08	40			26,978,725	26,374,582	5,146,066	14.4
PAM7	Bo5	LGC16_JH11	36			28,093,637	27,295,061	3,814,512	13.1
PAP2	Rui9	LGC_JH01	39.5		*SB/Sb*	30,670,661	29,778,253	5,165,706	19.6
PAP3	Gui3	LGC_JH04	53			24,020,182	23,073,781	3,728,456	19.1
PAP6	Bang3	LGC_JH07	40			30,685,360	29,937,298	6,284,714	11.0
PAP7	Bo5	LGC16_JH10	36			28,858,541	28,321,302	4,145,514	41.0

Table S2. Predicted functions, expression pattern, and locations of 81 differentially expressed genes in the antennae of three worker classes: monogyne *SB/SB* , polygyne *SB/SB* , and polygyne *SB/Sb*

We first mapped QC-passed reads onto the fire ant genome Si_gnH following the Tuxedo pipeline to generate the Tux-gene set. Of these, we selected the putative coding genes by comparing the expressed genes to the databse NCBInr. We subsequently analyzed DEGs by mapping QC-passed reads on the putative coding genes, that were expressed in the antennae, by Bowtie 2. We estimated gene expression level with RSEM and tested difference expression with EBSeq package in R

[a]The genes in this table (top to bottom) correspond to the those in the figure 1 (top to bottom). Genes with SINV prefix were annotated in the official gene set. Genes with XLOC prefix were generated by the Tuxedo pipeline

[b]The predicted functions of the genes with "SINV" prefixes have remained as annotated in the OGS. The predicted functions of the genes with "XLOC" prefixes were obtained by comparing transcript sequences against UniRef50 using blastx with an evalue threshold of e-20. Transposable elements were identified by comparing transcript sequences to the Repeat Masker database (Repbase, last accessed in May 2019) with the following criteria: similarity > 90% and transcript length coverage ≥ 50%. "Uncharacterized protein" was assigned to the genes of unknown predicted function from the above comparisons.

[c]From UniProt database

[d]The comparison having differential of the three pair-wise comparisons; M_BB = monogyne *SB/SB* , P_BB = polygyne *SB/SB,* P_Bb = polygyne *SB/Sb,* all different = differentially expressed in all three comparisons

[e]Differentially expressed genes were defined based on posterior probabilities of differential expression (PPDE) ≥ 0.95 in EBSeq

[f]Chromosome location of the genes based on the genome F genetic map (Wurm et al. (2011), Wang et al. (2013)). Linkage group 16 (LG16) is the social chromosome. "NA" indicates un-mapped scaffold. The genes located within the supergene boundaries indicated with "supergene" following Pracana et al. (2017) and Huang et al. (2018)

(*)Best hit was *Histone-lysine N-methyltransferase SETMAR-like* , which in insects are usually simply a *Mariner* transposon, but manual inspection revealed that it has low similarity to both the predicted gene and the *Mariner* transposon.

(**) *Integrase core domain protein* is characterized as chemical process in UniProt. It is widely found in viruses and transposons, thus, we classified it as transposable element (Rice et al. 1996; Malik and Eickbush 1999)

(***) Test with Tukey HSD showed that these genes were expressed more in P_Bb compared to both M_BB and P_BB

Gene[a]	Predicted function[b]	Biological process[c]	Comparison[d]	Log2_fold change	Up in	PPDE[e]	Linkage group[f]
XLOC_040838	Uncharacterized protein	NA	P_Bb vs. (P_BB, M_BB)	-0.75	*SB/SB*	1	NA
SINVm1_gene_00196	Nuclease harbi1-like	Transposable element	P_Bb vs. (P_BB, M_BB)	-0.8	*SB/SB*	1	NA
XLOC_003144	Transposable element tc1 transposase	Transposable element	P_Bb vs. (P_BB, M_BB)	-0.71	*SB/SB*	1	NA
XLOC_034528	Uncharacterized protein	NA	P_Bb vs. (P_BB, M_BB)	-2.36	*SB/SB*	1	LG11
XLOC_016888 (SiOR463)	Odorant receptor 22b	Odorant perception	P_Bb vs. (P_BB, M_BB)	-1.18	*SB/SB*	0.961	LG16, supergene
XLOC_000138	Uncharacterized protein	NA	M_BB vs. (P_Bb, P_BB)	4.29	Monogyne	0.955	NA
SINVm1_gene_10887	Uncharacterized protein	NA	M_BB vs. (P_Bb, P_BB)	2.56	Monogyne	1	NA
SINVm1_gene_12026	Polyadenylate-binding protein 1	Poly(A) binding	M_BB vs. (P_Bb, P_BB)	0.7	Monogyne	0.996	LG1
SINVm1_gene_01650	Glycerol-3-phosphate acyltransferase 3-like isoform 2	Chemical metabolism	M_BB vs. (P_Bb, P_BB)	0.67	Monogyne	1	LG10
SINVm1_gene_04716	Exonuclease 3 -5 domain-like-containing protein 1	Double-strand process	M_BB vs. (P_Bb, P_BB)	0.41	Monogyne	0.993	LG6
SINVm1_gene_04127	Udp-glucuronosyltransferase 2b30-like	Metabolism process	M_BB vs. (P_Bb, P_BB)	0.42	Monogyne	0.995	LG4
XLOC_001622	Uncharacterized protein	NA	M_BB vs. (P_Bb, P_BB)	0.85	Monogyne	1	NA
SINVm1_gene_03610	Alpha,alpha-trehalose-phosphate synthase	Biosynthetic process (chemical metabolism)	M_BB vs. (P_Bb, P_BB)	0.69	Monogyne	0.999	LG2 or LG4
SINVm1_gene_10899	Mariner 29	Transposable element	M_BB vs. (P_Bb, P_BB)	3.21	Monogyne	1	NA
SINVm1_gene_00676	Fatty acid synthase	Fatty acid metabolism (chemical metabolism)	M_BB vs. (P_Bb, P_BB)	2.85	Monogyne	0.975	NA
SINVm1_gene_00053	Transient receptor potential cation channel protein painless	Ion transportation	M_BB vs. (P_Bb, P_BB)	0.66	Monogyne	1	NA
XLOC_001842	Pol-like protein	Transposable element	M_BB vs. (P_Bb, P_BB)	1.07	Monogyne	0.972	NA
SINVm1_gene_07970	Agap000696-pa isoform 1	NA	M_BB vs. (P_Bb, P_BB)	0.8	Monogyne	0.975	LG3

SINVm1_gene_06423	Protein croquemort-like	Apoptosis	M_BB vs. (P_Bb, P_BB)	0.89	Monogyne	1	LG5
SINVm1_gene_03418	Uncharacterized mfs-type transporter c19orf28 homolog	Transporter	M_BB vs. (P_Bb, P_BB)	0.89	Monogyne	0.978	LG1
SINVm1_gene_14166	Acyl-delta desaturase-like	Fatty acid metabolism (chemical metabolism)	M_BB vs. (P_Bb, P_BB)	2.1	Monogyne	1	LG5
SINVm1_gene_02300	Pro-phenol oxidase subunit 2	Defense system (chemical metabolism)	M_BB vs. (P_Bb, P_BB)	2.02	Monogyne	0.982	LG12
SINVm1_gene_01163	Fatty acyl-reductase cg5065-like	Fatty acid metabolism (chemical metabolism)	M_BB vs. (P_Bb, P_BB)	1.21	Monogyne	0.993	LG2 or LG8
SINVm1_gene_02017	Guanylate cyclase 32e	cGMP biosynthesis	M_BB vs. (P_Bb, P_BB)	0.47	Monogyne	0.976	LG1
XLOC_043759	Uncharacterized protein	NA	M_BB vs. (P_Bb, P_BB)	0.79	Monogyne	0.997	NA
SINVm1_gene_12498	Atp-binding cassette sub-family g member 1	Transporter	M_BB vs. (P_Bb, P_BB)	1.02	Monogyne	0.993	LG6
SINVm1_gene_10405	Si:ch211- protein	NA	P_Bb vs. (P_BB, M_BB)	4.15	*SB/Sb*	1	NA
SINVm1_gene_00166	Transposase domain-containing protein	Transposable element	all different(***)			1	NA
SINVm1_gene_00401	Probable cytochrome p450 4aa1	Chemical metabolism	P_Bb vs. (P_BB, M_BB)	6.29	*SB/Sb*	1	NA
XLOC_042917	Copia 12	Transposable element	P_Bb vs. (P_BB, M_BB)	1.98	*SB/Sb*	1	NA
SINVm1_gene_13689	Uncharacterized protein	NA	P_Bb vs. (P_BB, M_BB)	1.61	*SB/Sb*	1	NA
XLOC_044145	Uncharacterized protein	NA	P_Bb vs. (P_BB, M_BB)	2.33	*SB/Sb*	0.993	NA
XLOC_000568	Uncharacterize protein	NA	P_Bb vs. (P_BB, M_BB)	4.12	*SB/Sb*	0.964	NA
SINVm1_gene_10777	Nuclease harbi1-like	Transposable element	P_Bb vs. (P_BB, M_BB)	1.06	*SB/Sb*	1	NA
SINVm1_gene_05326	Uncharacterized protein	NA	P_Bb vs. (P_BB, M_BB)	1.32	*SB/Sb*	1	LG16, supergene
XLOC_000436	Lambda-type exonuclease	Virus protein	P_Bb vs. (P_BB, M_BB)	1.81	*SB/Sb*	0.999	NA
XLOC_036229	Orf	Virus protein	P_Bb vs. (P_BB, M_BB)	0.9	*SB/Sb*	1	LG1
XLOC_000229	Gypsy 19	Transposable element	P_Bb vs. (P_BB, M_BB)	2.46	*SB/Sb*	1	NA
XLOC_023347	Uncharacterized protein	NA	all different(***)			1	LG3
SINVm1_gene_00322	Si:dkey- protein	NA	P_Bb vs. (P_BB, M_BB)	3.66	*SB/Sb*	1	NA
XLOC_001413	Uncharacterized protein	NA	P_Bb vs. (P_BB, M_BB)	1.66	*SB/Sb*	1	NA
XLOC_002426	Uncharacterized protein	NA	P_Bb vs. (P_BB, M_BB)	0.84	*SB/Sb*	0.982	NA
SINVm1_gene_09757	Upf0439 protein c9orf30 homolog	Cell adhesion, host-virus interaction	P_Bb vs. (P_BB, M_BB)	2.64	*SB/Sb*	0.999	LG11
SINVm1_gene_09325	Integrase core domain protein	Transposable element(**)	P_Bb vs. (P_BB, M_BB)	7.39	*SB/Sb*	1	NA
SINVm1_gene_05175	Nadh dehydrogenase	Catalysis process (chemical metabolism)	P_Bb vs. (P_BB, M_BB)	4.13	*SB/Sb*	1	LG16, supergene
SINVm1_gene_02111 (SiOBP12b')	Odorant binding protein	Odorant perception	P_Bb vs. (P_BB, M_BB)	4.64	*SB/Sb*	1	LG16, supergene
SINVm1_gene_10721	Deoxyribonuclease tatdn1	DNA process	M_BB vs. (P_Bb, P_BB)	-3.85	Polygyne	0.959	NA
XLOC_041741	Ben domain-containing protein 6-like	Neurogenesis, transcription regulation	P_Bb vs. (P_BB, M_BB)	2.58	*SB/Sb*	0.987	LG1
XLOC_001664	Pol protein	Transposable element	P_Bb vs. (P_BB, M_BB)	1	*SB/Sb*	0.959	NA
XLOC_003527	Multiple epidermal growth factor-like domains protein 8-like	Signaling pathway	P_Bb vs. (P_BB, M_BB)	4.51	*SB/Sb*	1	NA
SINVm1_gene_00935	Nuclease harbi1-like	Transposable element	P_Bb vs. (P_BB, M_BB)	2.75	*SB/Sb*	1	NA
SINVm1_gene_00321	Uncharacterized protein	NA	P_Bb vs. (P_BB, M_BB)	3.53	*SB/Sb*	1	NA
XLOC_036812	Uncharacterized protein	NA	P_Bb vs. (P_BB, M_BB)	2.6	*SB/Sb*	1	NA
XLOC_001846	L2-1 sinv	Transposable element	P_Bb vs. (P_BB, M_BB)	1.86	*SB/Sb*	1	NA
XLOC_001337	Uncharacterized protein	NA	P_Bb vs. (P_BB, M_BB)	2.08	*SB/Sb*	1	NA
SINVm1_gene_05144	Coiled-coil domain-containing protein 142	NA	P_Bb vs. (P_BB, M_BB)	2.24	*SB/Sb*	1	LG16, supergene
SINVm1_gene_13984	Tyrosine-protein kinase transmembrane receptor ror	NA	P_Bb vs. (P_BB, M_BB)	0.65	*SB/Sb*	0.99	LG16, supergene
XLOC_041027	Uncharacterized protein	NA	P_Bb vs. (P_BB, M_BB)	1.95	*SB/Sb*	1	NA

SINVm1_gene_05176	Ubiquitin-like domain-containing ctd phosphatase 1	Chemical metabolism	P_Bb vs. (P_BB, M_BB)	0.93	*SB/Sb*	1	LG16, supergene
XLOC_000345	Uncharacterized protein	NA	P_Bb vs. (P_BB, M_BB)	0.89	*SB/Sb*	1	NA
XLOC_034835	Uncharacterized protein	NA	M_BB vs. (P_Bb, P_BB)	-0.76	Polygyne	0.984	NA
SINVm1_gene_10049	Iduronate 2-sulfatase	Chemical metabolism	M_BB vs. (P_Bb, P_BB)	-0.75	Polygyne	0.992	LG2
XLOC_032988	Gypsy 7	Transposable element	M_BB vs. (P_Bb, P_BB)	-1.7	Polygyne	0.997	LG15
XLOC_030870	Aaa atpase	ATP-binding	M_BB vs. (P_Bb, P_BB)	-1.17	Polygyne	0.991	LG11
SINVm1_gene_01363	Cop9 signalosome complex subunit 2	Cellular and developmental process	M_BB vs. (P_Bb, P_BB)	-1.37	Polygyne	0.97	LG7 or LG11
XLOC_042434	Dnaj homolog subfamily b member 6-like	Protein folding	M_BB vs. (P_Bb, P_BB)	-0.46	Polygyne	1	NA
XLOC_002577	Pol protein	Transposable element	M_BB vs. (P_Bb, P_BB)	-0.87	Polygyne	0.999	NA
SINVm1_gene_04713	Ribosomal rna-processing protein 7 homolog a-like	rRNA process	M_BB vs. (P_Bb, P_BB)	-0.52	Polygyne	1	LG6
SINVm1_gene_00238	Esterase fe4-like	Chemical metabolism	M_BB vs. (P_Bb, P_BB)	-0.71	Polygyne	0.974	LG2
XLOC_020263	Cugbp elav-like family member 2 isoform x7	RNA binding	M_BB vs. (P_Bb, P_BB)	-0.5	Polygyne	1	LG1
SINVm1_gene_10481	Nuclease harbi1-like	Transposable element	M_BB vs. (P_Bb, P_BB)	-0.75	Polygyne	1	NA
XLOC_012652	Uncharacterized protein	NA	M_BB vs. (P_Bb, P_BB)	-1.04	Polygyne	0.966	LG1
XLOC_011724	Uncharacterized protein (*)	NA	M_BB vs. (P_Bb, P_BB)	-0.62	Polygyne	0.99	LG3
XLOC_027187	Whirlin-like isoform 2	Sensory perception (light or sound)	M_BB vs. (P_Bb, P_BB)	-0.35	Polygyne	0.994	LG13
SINVm1_gene_14059	Uncharacterize protein cg7029-pc	NA	M_BB vs. (P_Bb, P_BB)	-0.26	Polygyne	0.983	LG5
XLOC_006396	Uncharacterized protein	NA	M_BB vs. (P_Bb, P_BB)	-0.67	Polygyne	0.956	LG7 or LG11
SINVm1_gene_04300	Ankyrin repeat and lem domain-containing protein 1	DNA damage and repair	M_BB vs. (P_Bb, P_BB)	-0.7	Polygyne	0.986	LG3
XLOC_003605	Uncharacterized protein	NA	M_BB vs. (P_Bb, P_BB)	-1.86	Polygyne	1	NA
XLOC_003265	Uncharacterized protein	NA	M_BB vs. (P_Bb, P_BB)	-1.92	Polygyne	1	NA
XLOC_041335	Uncharacterized protein	NA	M_BB vs. (P_Bb, P_BB)	-1.8	Polygyne	0.995	NA
SINVm1_gene_09125	Nuclease harbi1-like	Transposable element	M_BB vs. (P_Bb, P_BB)	-2.25	Polygyne	0.986	LG10

Huang Y, Dang VD, Chang N, Wang J. 2018. Multiple large inversions and breakpoint rewiring of gene expression in the evolution of the fire ant social supergene. *Proc. R. Soc. B* 285(1878):20180221

Malik HS, Eickbush TH. 1999. Modular evolution of the integrase domain in the Ty3/Gypsy class of LTR retrotransposons. *J. Virol* . 73(6):5186–5190.

Pracana R., Priyam A, Levantis I, Nichols RA, Wurm Y. 2017. The fire ant social chromosome supergene variant *Sb* shows low diversity but high divergence from *SB. Mol. Ecol.* 26(11):2864–2879

Rice P, Craigie R, Davies DR. 1996. Retroviral integrases and their cousins. *Curr. Opin. Struct. Biol.* 6(1):76–83.

Wang J, Wurm Y, Nipitwattanaphon M, Riba-Grognuz O, Huang Y-C, Shoemaker D, Keller L. 2013. A Y-like social chromosome causes alternative colony organization in fire ants. *Nature* 493 (7434) : 664–668

Wurm Y, Wang J, Riba-Grognuz O, Corona M, Nygaard S, Hunt BG, Ingram KK, Falquet L, Nipitwattanaphon M, Gotzek D, et al. 2011. The genome of the fire ant *Solenopsis invicta. Proc. Natl. Acad. Sci.* 108(14):5679–5684

Table S3. ***OBP12*** **locus allele-specific expression is prominently from the** ***SiOBP12b'***

Data called by GATK based on mapping a pool of four polygyne SB/Sb antennal RNA-seq libraries to the gnH genome assembly. *SiOBP12* is located on scaffold Si_gnH.scaffold00042. Only the SNP/indel sites in the exons are considered.

[a]Phred-scaled probability (reference/alternative) that a polymorphism exists, based on the sequencing data

[b]Number of reads supporting the reference (*SiOBP12B* , B_num) or alternate (*SiOBP12b'* , b'_num) alleles

*Poor mapping caused by the region having high SNP and indel density between the *SiOBP12* and *SiOBP12b'* (not included in the total)

**Binomial exact test

						Excluded reads due to poor mapping*	
**Position**	**Reference**	**Alternative**	**Quality[a]**	**B_num[b]**	**b'_num[b]**	**B_num[b]**	**b'_num[b]**
440686	A	G	8481.77	19	720		
440691	C	T	8561.77	22	718		
440703	A	G	9167.77	19	781		
440759	G	A	6212.77	12	582		
441042	C	T	NA			6	19
441054	C	G	298.77			6	18
441062-441068	TTTTTCA	T	247.73			6	12
441071	A	T	172.77			6	12
441072	T	C	130.77			6	12
441074	A	AGTC	121.73			6	12
441078	G	T	130.77			6	12
441082	C	T	6615.77			7	11
441083	T	A	6660.77			6	18
441094	T	C	7808.77	6	591		
441126	T	C	10272.77	3	585		
441656	G	C	8464.77	1	35		
441657	G	A	8377.77	0	34		
441664	C	T	421.77	0	17		
441667	G	T	241.84	0	13		
441984	T	G	6600.77	2	274		
441985	G	A	6600.77	1	270		
442005	T	A	6338.77	2	247		
442027	G	A	5009.77	0	185		
			**Total reads**	**87**	**5052**		
			***P*-value**	**< 2.2e-16 ****			

Table S4. *SiOBP12* copy number variation analysis

[a]Family data are from Wang et al. 2013, except *SB* of family 8 which is from Wurm et al. 2011

[b]500 bp sliding windows with an overlap of 250 bp; the length of *SiOBP12B* cDNA (HQ853360) is 522 bases

[c]log2 ratio of reads mapped onto *Sb* and *SB* genome, normalized by library size

[d]Average of log2 ratio of mapped read for each window (window_log2) and for whole gene (gene_log2)

	Window		Raw count (reads)				
Family[a]	From[b]	To[b]	*Sb* genome	*SB* genome	log2-fold[c]	P-value	window_log2[d]
1	1	500	135	84	0.57	6.67E-04	1.01
2	1	500	134	68	1.13	1.65E-09	
3	1	500	113	74	0.54	3.62E-03	
4	1	500	122	58	0.97	2.53E-06	
5	1	500	153	71	1.09	1.02E-07	
6	1	500	138	46	1.17	1.52E-08	
7	1	500	99	50	0.99	3.60E-07	
8	1	500	450	29	1.62	6.82E-35	
1	251	522	137	70	0.85	2.03E-06	1.08
2	251	522	96	69	0.63	1.84E-04	
3	251	522	79	52	0.53	4.01E-03	
4	251	522	97	46	0.97	2.37E-06	
5	251	522	112	57	0.96	1.46E-06	
6	251	522	100	32	1.22	4.69E-09	
7	251	522	87	42	1.05	9.03E-08	
8	251	522	258	9	2.41	2.24E-50	
1	501	522	68	36	0.79	6.25E-06	0.95
2	501	522	34	37	0.05	0.40	
3	501	522	39	19	0.93	3.27E-06	
4	501	522	37	13	1.35	7.06E-10	
5	501	522	44	24	0.85	1.00E-05	
6	501	522	29	19	0.19	0.12	
7	501	522	39	23	0.76	3.32E-05	
8	501	522	128	3	2.73	4.13E-57	
							1.01 gene_log2[d]

Wang et al. 2013. A Y-like social chromosome causes alternative colony organization in fire ants. *Nature* 493(7434):664–668.
Wurm et al. 2011. The genome of the fire ant *Solenopsis invicta* . *Proc. Natl. Acad. Sci* . 108(14):5679–5684.

Table S5. Number of reads of associated with *SiOBP12B*, *SiOBP12b*, and *SiOBP12b′* in the 454 RNA sequencing data

[a]Total reads aligning to the first 80 nucleotides of *SiOBP12B* (HQ853360) starting from the 1st coding nucleotide

[b]Reads containing a T nucleotide at the 46th site (*SiOBP12B* allele)

[c]Reads containing a C nucleotide at the 46th site (*SiOBP12b′*)

[d]Reads containing a 17-nucleotide insertion at the 74th site (*SiOBP12b* allele)

Dataset	Colony form	Total[a]	*SiOBP12B*[b]	*SiOBP12b′*[c]	*SiOBP12b*[d]
SRR060011	monogyne	20	20	0	0
SRR060012	polygyne	32	23	8	1
SRR060013	polygyne	4	3	1	0

Table S6. List of primers used in this study

[a]This study unless otherwise noted

(*)Detect *SiOBP12B* , *SiOBP12b* , and *SiOBP12b'*

Target gene	Primer name	Assay	Primer sequence (5' - 3')	Reference[a]	Species
SiOBP12 (*)	OBP12-F227	qRT-PCR, 3'RACE	ATGGCTGCTTTATGGCGTGTTT		*Solenopsis invicta*
	OBP12-R302	qRT-PCR, 5'RACE	GCAATAACTTCATCTTTAATGTTGGTTCCT		
	OBP12-F227R	nested 5'RACE, sequence trace assay	AAACACGCCATAAAGCAGCCAT		
	OBP12-R302R	nested 3'RACE	AGGAACCAACATTAAAGATGAAGTTATTGC		
	OBP12Bb'-5'UTR	sequence trace assay	TAAAGATATAAATTGCACAAACATC		
	OBP12Bb'-3'UTR	sequence trace assay	ATATTTAACTGTGTTTATTCCGTCAGC		
SiOBP12b'	raceOBP12.5UTR.F	PCR	GTTCTACAAGGAGTGTCGATCTACTTGTCCTCG		
	raceOBP12.3UTR.R	PCR	CCGTATATTTAACTGTGTTTATTCCGTCAGC		
Gp-9	B-F312	qRT-PCR	TCCTGGTTGAGCACCTGTTTCC		
	B-R422	qRT-PCR	GGTTGCGTCATGCAATGTTTGT		
XLOC_021041	041FC171126	qRT-PCR	CTGAAATGGCGAAGAGAGTATTTGGACTTGG		
	041RC171126	qRT-PCR	CACTTATATGGTCGTCATACCTTGAGTTTGTTTCC		
XLOC_020416	20416F171108B	qRT-PCR	GCTGTATGGAATAAAGGAAGTAAACGGTG		
	20416R171108B	qRT-PCR	TTTGTCATTCTACTCAAAGTATAATTTATTTGCGAG		
XLOC_020357	20357F2	qRT-PCR	CAATGTATCAATGTGGGAAAAGGGT		
	20357R4	qRT-PCR	CTCCTCAAGTGTCCTAACTCGTCC		
SgOBP12	SgOBP12_F85	qRT-PCR	CAAGAAGAAGTAAAGATCAAGCAACGAA		*Solenopsis geminata*
	SgOBP12_R167	qRT-PCR	GTCGGACCGTACAAGTCACTTTCATTTAT		
Elongation factor 1α	new_ELA_F	qRT-PCR	GGAATTGCGCCGTGGATATG		*Solenopsis invicta* *Solenopsis geminata*
	new_ELA_R	qRT-PCR	CCGTTGCTGATTTGACCAGG		
Elongation factor 1b	ef1-betaF	qRT-PCR	TGAAGACCGATAAGGGCA	Cheng et al. (2013)[1]	
	ef1-betaR	qRT-PCR	TCGTCCGAACCAAAGAGA	Cheng et al. (2013)[1]	
Commercial primers	LongUP	3'RACE	CTAATACGACTCACTATAGGGCAAGCAGTGGTATCAACGCAGAGT	Clontech[2]	
	ShortUP	3'RACE	CTAATACGACTCACTATAGGGC	Clontech[2]	
	5'RACE AAP	5'RACE	GGCCACGCGTCGACTAGTACGGGIIGGGIIGGGIIG	Invitrogen[3]	
	AUAP	5'RACE	GGCCACGCGTCGACTAGTAC	Invitrogen[3]	

[1]Cheng D, Zhang Z, He X, Liang G. 2013. Validation of reference genes in *Solenopsis invicta* in different developmental stages, castes and tissues. *PLoS ONE* 8(2): e57718. doi:10.1371/journal.pone.0057718

[2]SMART RACE cDNA Amplification Kit (634914, Clontech)

[3]5′ RACE System V2.0 kit (18374-058, Invitrogen)

Table S7. IDs of the four *SB* PacBio raw reads that cross Gap1 and Gap2 on scaffold Si.gnH_scaffold00042

The *SiOBP12b'* region corresponds to a region in Si.gnH_scaffold00042 with two gaps, Gap 1 and Gap 2. Examination of the *SB* raw PacBio sequencing reads revealed four reads that crossed the two gaps.

Gap	PacBio raw read IDs
Gap 1	m150822_084835_42180_c100834262550000001823178012191565_s1_p0/60739/0_12931
	m150611_053507_42180_c100817392550000001823183010291503_s1_p0/70538/15563_34997
	m151014_172658_42180_c100833992550000001823178012191537_s1_p0/109395/25973_36318
Gap 2	m150611_053507_42180_c100817392550000001823183010291503_s1_p0/103132/2117_14831
