# Supplementary material for: Has gene expression neofunctionalization in the fire ant antennae contributed to queen discrimination behavior?
Source: Ecol Evol. 2019 Oct 29;9(22):12754–66. doi: 10.1002/ece3.5748 (PMC6875580; doi:10.1002/ece3.5748)
Supplement: Supplementary file 3 [file ECE3-9-12754-s003.pdf]

Supplementary materials

Has gene expression neofunctionalization in the fire ant antennae contributed to queen discrimination behavior?

Viet-Dai DANG[*,†,‡,§], Amir B. COHANIM[**], Silvia FONTANA[*,†,‡], Eyal PRIVMAN[**], John WANG[*]

[*]Biodiversity Research Center, Academia Sinica, Taipei, Taiwan

[†]Biodiversity Taiwan International Graduate Program, Biodiversity Research Center, Academia Sinica

[‡]Department of Life Science, National Taiwan Normal University, Taipei, Taiwan.

[§]Department of Zoology, Southern Institute of Ecology, Hochiminh, Vietnam

[**]Department of Evolutionary and Environmental Biology, Institute of Evolution, University of Haifa, Haifa 3498838, Israel

Corresponding author: John WANG

Biodiversity Research Center, Academia Sinica, Taipei 115, Taiwan

johnwang@gate.sinica.edu.tw

TEL: +886 2 27871582

FAX: +886 2 27871587

MATERIALS AND METHODS

Ant colony collection, maintenance and genotyping

Red imported fire ant, *S. invicta,* colonies were collected in Taoyuan County, Taiwan. Ant colonies were initially assessed as monogyne or polygyne based on the shape of the ant mound, worker size, and the distance between mounds in the field. Subsequently the social form of each colony was confirmed by the number of queens in a colony and *Gp-9* genotyping using PCR/RFLP on pooled DNA from at least ten workers (Krieger and Ross 2002). We maintained the colonies under standard conditions (Jouvenaz et al. 1977). Ants were sampled at least two to three months after collection from the field.

Two monogyne colonies of tropical fire ant, *S. geminata*, were collected in Taichung City, Taiwan. After collection, we maintained them in the lab in the same conditions as *S. invicta*, except with the addition of seeds used as bird food.

Sample and tissue collection

S. invicta:

Because the queen discrimination behavior could happen at the nest entrance, we assumed that new queens would first encounter foraging workers before entering the nest (Gotzek and Ross 2008). Therefore, we used a droplet of honey to attract foragers and sampled medium-sized individuals (ca. 1.5 mm in length). We sampled 13 bio-replicates (figure S1A-B); a bio-replicate is composed of one pair of a monogyne and a polygyne colony. These colonies were collected from the field on the same day and sampled two to three months later, also on the same day. Each bio-replicate consisted of three groups of sampled workers: monogyne *SB/SB* (M_BB), polygyne *SB/SB* (P_BB), and polygyne *SB/Sb* (P_Bb). We used four bio-replicates for RNA sequencing,

with each sample containing 36 to 55 antennae pairs. The remaining nine were used for qRT-PCR experiments: preliminary tests, RNA-seq candidate gene validation, and OBP expression localization in different body parts. Each qRT-PCR experiment consisted of three bio-replicates. Samples for the qRT-PCR experiments were derived from 20 to 24 antennae pairs or 15 units of a body part (heads, thoraces, or abdomens). To prepare samples for sequence trace analysis of *SiOBP12* on different worker body parts, we sampled 10 to 11 *SB/Sb* workers from three additional polygyne colonies (figure S1C).

To obtain many antennae of the same genotype and social form for pooling, we first determined the *Gp-9* genotype of every individual (figure S1A). To do so, workers were decapitated one-by-one on a pre-chilled -20°C metal block sitting on ice. Worker heads were placed individually in 5 µl cold RNAlater ICE (AM7023, ThermoFisher) in a numbered 0.2 ml PCR-tube and temporarily stored in liquid nitrogen. Worker bodies were put in 2 ml tubes containing 50 µl QuickExtract solution (QE09050, Epicenter) and processed for *Gp-9* genotyping. The processing of each individual, from sampling to head storage in liquid nitrogen was less than a minute. We sampled 96 individuals per polygyne and 50 individuals per monogyne colony each time. The head samples were stored in a -80°C freezer for subsequent antennae dissection. We sampled polygyne workers until we obtained at least 45 individuals of each genotype. We dissected the antennae of workers of the same genotype on a metal block sitting on ice, under a dissecting microscope (Leica, 20X magnification). We used a clean sheet of Kimwipe tissue to blot away any remaining RNAlater ICE on the samples before placing them into 200 µl cold TRIzol (15596018, Invitrogen). To minimize RNA degradation, we dissected the antennae of at most five heads at once. To minimize cross-sample contamination, we cleaned the metal block and forceps with RNase-Zap after each dissection. Antennae were stored in a -80°C freezer until RNA extraction.

To prepare samples of different body parts of the workers, we followed the same process as antennal preparation. To genotype individuals for the qRT-PCR assays on the head, thorax, and abdomen, we genotyped the antennae (figure S1B). To genotype individuals for the sequence trace profile analysis on the antenna, head, and body (i.e., thorax-abdomen), we genotyped 2 random legs (figure S1C).

S. geminata:

We sampled foraging workers and then dissected the antennae, heads, thoraces, and abdomens as described for *S. invicta*. We collected the head, thorax, and abdomen from 20 or 26 individuals. For the antennae samples, we collected 99 or 108 pairs.

RNA extraction

We extracted RNA using the Direct-zol RNA MiniPrep kit (R2050, ZymoResearch) following a modification of the manufacturer's instructions (Zhang et al. 2013). Frozen antennae in 200 μl Trizol were homogenized using a FastPrep-24 bead homogenizer (MP Biomedicals). Tissue destruction was checked under a microscope. If extra homogenization was required, we re-froze the sample in liquid nitrogen and repeated the homogenization (three times was the maximum needed). After the tissue was completely homogenized, we added an additional 400 μl Trizol into each sample, followed by vortexing for 10 secs and incubation at room temperature for 15 minutes. Samples were then phase separated with 120 μl chloroform. The aqueous phase was kept for RNA purification using the Direct-zol column with on-column DNase I treatment. RNA was eluted once in 25 μl (*S. invicta*) or 36 μl (*S. geminata*) RNase-free water.

RNA extraction from non-antennal tissues followed the same process of antennal RNA extraction, except that the total volume of Trizol was 1 ml and that of chloroform was 200 μl. *S.*

invicta samples were eluted twice with 25 μl RNase-free water (50 μl total). For *S. geminata*, the thorax samples were eluted once with 36 μl; the head and abdomen samples were eluted twice with 30 μl Rnase free water (total 60 μl).

The integrity and quantity of RNA samples were checked using a Nanodrop 1000 and a Bioanalyzer 2100.

RNA amplification and sequencing

To obtain sufficient cDNA for sequencing, we amplified the cDNA from the antennal RNA samples. We used two protocols.

In method one, we amplified 25 ng of each RNA sample (four bio-replicates of three worker classes, i.e., 12 amplifications) using the Nugen Ovation V.2 kit (M01206, NuGEN Technologies), following the manufacturer's instructions. These amplified samples were sequenced in two ways. In an initial pilot experiment, we pooled the four amplified bio-replicates (200 ng amplified cDNA per sample) by worker class prior to library preparation (i.e., three libraries, 800 ng of amplified-cDNA-then-pooled per library) and sequencing. For DEG analysis, we sequenced each of the 12 amplified samples separately in a 12-plex fashion on two lanes. In both cases, each sample was amplified no more than 10 additional cycles during TruSeq DNA sample preparation.

In method two, we pooled 200 ng of total RNA from each of the three bio-replicates of each worker class (i.e., three pooled RNA samples). These pooled samples were polyA(+)-RNA selected, amplified using the SMARTer Stranded RNA-Seq kit, and then sequenced directly (Clontech; at the High Throughput Genomics Core at the Biodiversity Research Center, Academia Sinica, Taiwan [HTGC BRC]). Sequencing results in method two (pooled-total-RNA-then-

amplified) were used to compare to the sequencing results of the samples in method one (amplified-cDNA-then-pooled) to decide the bioinformatics approach for differential gene expression analysis.

All samples were sequenced on the Illumina HiSeq 2500 platform with a 150 bp paired-end protocol (HTGC BRC).

Quantitative real time PCR (qRT-PCR):

We designed primers for the qRT-PCR assays as previously described (Thornton and Basu 2011). In brief, primers were designed using Primer3plus (table S6). The primers were chosen if they spanned two exons or the forward and reverse primers of each pair were located on two different exons. We then used Beacon Designer (Premier Biosoft International) and mFold (Zuker 2003) to check for cross dimers, self-dimers, hairpins, and secondary structure of each amplicon ($\Delta G > -3$), all of which can affect fluorescence signal intensity.

The 1st strand cDNA samples were prepared using SuperScript III (18080044, Invitrogen) following the manufacturer's instructions, with the addition of random primers (N410-01, Protech) and RNaseOUT (10777019, Invitrogen).

We performed qRT-PCR experiments using KAPA SYBR FAST qPCR Master Mix (2X) ABI Prism (KK4603, KapaBiosystems) following the manufacturer's instructions. The qRT-PCR assays were conducted on an ABI QuantStudio 5 system (ThermoFisher), following the default program (initial denaturation for 10 minutes at 95°C; 32-35 cycles of denaturation for 15s at 95°C; final annealing and elongation for 1 min at 60°C). The number of PCR cycles was decided based on the RNA concentrations of the samples in a batch. A dissociation step was added to test for non-specific amplification via dissociation curves.

To compare the expression level of the target genes in different samples, the expression of the target genes in a specific sample were calculated as the difference in cycle number of the genes and the average of the cycle numbers of the two internal control genes of that sample (ΔCt). To display the relative expression level of the target genes in different worker classes in a specific tissue, we compared the ΔCt of that gene in each bio-replicate to the average of the ΔCt of that gene in that tissue of the monogyne *SB/SB* worker (M_BB). We used the Tukey HSD test to test differential expression.

RNA-seq data processing

For all 12 cDNA libraries (Nugen amplified), we controlled the quality of the sequence reads by retaining those passing a base-quality threshold of 20 and minimum length of 30 bases using cutadapt v1.16 (Martin 2011). After, we examined the read duplication rate within the libraries by mapping these filtered reads onto the published genome assembly of *S. invicta* (version Si_gnH; NCBI accession AEAQ00000000), marking duplicates with samtools markdup, and then counting duplicates with samtools flagstat (table S1). The duplication rates (11%-41%) are comparable to other studies (Shanker et al. 2015; Song et al. 2018).

For the RNA-seq analysis we considered two potential reference gene sets: "Tux-gnH", a newly assembled gene set following the Tuxedo Suite method (Trapnell et al. 2012) for Si_gnH and "OGS", the official gene set that was published with this assembly (15,589 genes) (Privman et al. 2018). The Tux-gnH gene set was derived as follows. We used TopHat v2.0.11 to map the quality-controlled reads from all 12 libraries onto Si_gnH with the guidance of the OGS (i.e., -G). Subsequently, we ran Cufflinks v2.2.1 to assemble a gene set for each library followed by Cuffmerge v1.0.0 to generate Tux-gnH, resulting in 44,521 putative genes. Comparing to the OGS

using Cuffcompare v2.2.1, the Tux-gnH gene set has 15,116 genes (34%) that were annotated. The remaining 29,405 putative genes include protein coding genes, non-coding RNAs, and likely some false-positives since cDNA amplification can be over sensitive (Sun et al. 2013). The raw and processed data are available under NCBI accession GSE126684.

In an initial analysis of the two pilot amplified RNA-seq datasets (see above), we found that using the OGS produced in a greater overlap of the differentially expressed gene sets among the three sample groups (M_BB, P_BB, and P_B) compared to using Tux-gnH. This difference may be due to the effect of non-coding regions (e.g., introns) on the mapping results (Tux-gnH gene models retain introns, whereas gene models are spliced in the OGS gene set). Based on this observation, we decided to use the OGS gene set as the reference for the full analysis, but updated it to include newly annotated genes discovered in Tux-gnH. We included only those transcripts with protein coding potential, defined here as those encoding peptides with an open reading frame of at least 50 amino-acids and having similarity to genes in the NCBI non-redundant reference (NCBI nr) (Bethesda (MD): National Library of Medicine (US)). This added 4,114 genes to the OGS – for a total of 19,230 putative protein coding genes ("OGS-plus") (sequences in Dryad). Among these, 628 genes are located in the supergene, which is defined following the genetic map (Wang et al. 2013), the right supergene boundary (Huang et al. 2018), and five additional scaffolds (Pracana et al. 2017).

To identify differentially expressed genes (DEGs) on the full dataset of 12 samples, we first mapped the quality-controlled reads onto OGS-plus with Bowtie v.2.2.6 (Langmead and Salzberg 2012; Langmead et al. 2019). Next, we used RSEM v1.3.0 (Li and Dewey 2011) to estimate gene expression levels from the mapped reads. Finally, we identified DEGs using the EBSeq package v1.2.0 (Leng et al. 2013) (figure S1D). Each library was normalized by library

size using quantile normalization (Sizes = QuantileNorm). Visualization of the expression patterns (heatmap and plots) was based on Transcripts Per Million (TPM).

Variant calling at *SiOBP12*

The expression of *SiOBP12* in the *SB/Sb* worker antennae could be due to the gene expression of the original *SiOBP12B* or the paralog *SiOBP12b'*. To estimate the expression of *SiOBP12* and *SiOBP12b'*, we used GATK for variant-calling from the RNA-seq reads that were mapped onto the *SiOBP12* locus (Van der Auwera et al. 2002; McKenna et al. 2010; DePristo et al. 2011). Due to sequence divergence between *SiOBP12b'* and *SiOBP12* causing poor mapping quality, SNP calling for single *SB/Sb* biological samples was inaccurate. To increase the mapping read depth in the region, we merged all filtered reads of the four bio-replicates of *SB/Sb* antennal cDNA libraries prior to mapping this pool onto the Si_gnH genome assembly. The variants at this locus were subsequently called based on the mapping results of this *SB/Sb* pool.

Re-examination of the *SiOBP12* sequence from the fire ant EST library and other RNA sequencing data

The sequence of the *SB* allele of *SiOBP12,* (hereafter *SiOBP12B,* NCBI RefSeq HQ853360 (Gotzek et al. 2011)) contains an intact open reading frame but we found that *SiOBP12B* in the Si_gnH assembly has an early stop codon caused by a C-to-T substitution at the 46th nucleotide from the start codon (see also Pracana et al. 2017). To check whether *SiOBP12B* has an allele which is intact, we re-examined the EST library (consisting of multiple castes and stages from both monogyne and polygyne colonies) (Wang et al. 2007) and three RNA-seq dataset generated from a Roche 454 FLX machine (Wurm et al. 2009) which were used to construct the sequence of

HQ853360. We identified ESTs and RNA sequencing reads that may have contributed to the *SiOBP12B* sequence by blastn comparisons (Altschul et al. 1990) and counted the number of reads which contained the C or T nucleotide (at position 46th for *SiOBP12B*) or the 17-nucleotide insertion (at position 74th for *SiOBP12b*).

Structure analysis of SiOBP12 proteins

To examine if the amino acid differences between SiOBP12B, SiOBP12b, SiOBP12b', and SgOBP12 may be potentially functionally important, we compared their predicted protein sequences to Gp-9 (NCBI RefSeq Q8WP90) and the pheromone binding protein (PBP) in *Bombyx mori* (NCBI RefSeq NP_001037494). SiOBP12b' was translated from RACE cDNA sequence obtained in this study. SiOBP12B (Wurm et al. 2011), SiOBP12b (Wang et al. 2013), and SgOBP12 (Dryad) were translated from their respective genomic sequences. We aligned these six protein sequences using MEGA7 (Kumar et al. 2016). The helices and the residues, which are likely to be involve in the bombykol binding pocket and pH-sensitive conformation change, were adopted from previous studies (Sandler et al. 2000; Krieger and Ross 2005).

Copy number variation (CNV) analysis

CNV analysis was conducted in an independent study (Fontana et al., in review). In brief, we compared whole genome sequencing reads from eight pairs of sons (1 *SB* and 1 *Sb*, ant males are haploid) from unrelated *SB/Sb* queens (Wurm et al. 2011; Wang et al. 2013). We first generated a comprehensive, non-redundant gene list containing 27,279 *S. invicta* genes by combining all known *S. invicta* transcriptome from the OGS, NCBI and other available RNA-seq datasets. Redundancies among datasets (blastn, percent identity ≥ 95% and bitscore ≥ 200) were discarded.

Redundancies within datasets (this is for the RNA-seq derived transcripts) were filtered using CD-HIT with sequence identity threshold 95% (Li and Godzik 2006; Fu et al. 2012).

Each of the 16 datasets was aligned against this transcriptome reference using BWA mem v.0.7.12 (Li and Durbin 2009). To determine the copy number variation for each gene between haploid *SB* and *Sb* brothers, DNA read depths obtained from the BWA alignment were compared using CNV-seq (Xie and Tammi 2009) with window-size of 500 bp and step-size of 250 bp. For each overlapping window, we calculated “log2-fold(+1)” values normalized for different read depths using the formula: *log2-fold(+1) = log2(((Sb read count +1) / total Sb reads)/((SB read count +1) / total SB reads)).*

SiOR463 **deletion examination in** ***S. invicta SB, Sb,*** **and** ***S. geminata*** **genomes**

To check the deletion of the odorant receptor *SiOR463* (*XLOC_016888*), we mapped low-coverage sequenced data from 14 males (7 pairs of 1 *SB* and 1 *Sb* brothers) (Wang et al. 2013) onto contig 000892F of a PacBio *SB*-genome assembly (version t4p) and contig 000200F of a PacBio *Sb*-genome assembly (version t2p) using BWA mem (Li and Durbin 2009) (contig sequences in Dryad). The location of the OR cluster (i.e., *SiOR163*, *SiOR463* and *SiOR462*) was identified by blastn (Altschul et al. 1990) with the mRNA sequence of *SiOR163* (NCBI RefSeq XM_026133769) to the PacBio genomes of *S. invicta SB*, *Sb*, and *S. geminata* (blast results in Dryad). Subsequently, these regions were aligned and visualized with Mauve (Darling et al. 2004).

Ascertaining the presence or absence of ***SiOBP12b’*** **in the** ***S. invicta SB*** **and** ***S. geminata*** **genomes**

To examine whether the *S. invicta SB* and the monogyne *S. geminata* genomes have the *SiOBP12b'* gene or its remnants, we conducted a blastn comparison against the *S. invicta SB* and *S. geminata* genomes using contig 000102F of the *Sb* genome (version t2p) on which *SiOBP12b'* is located.

In the *SB* PacBio genome (version BigBt4p), we found that the *SiOBP12b'* region is located in a gap between contigs 001445F and 000408F of the BigBt4p genome (figure S8). In Si_gnH, the *SiOBP12b'* region corresponds to scaffold00042 (Si_gnH.scaffold00042), which itself has two gaps, Gap 1 and Gap 2. Gap 1 is surrounded by TA microsatellite repeats, while Gap 2 seems to be a unique sequence. We noticed that the 3'-end of the BigBt4p 000408F contig did not match Si_gnH.scaffold00042. Detailed investigation into the region revealed an assembly error at a repeat. Therefore, we clipped off the 3'-end of BigBt4p 000408F at the error site. To determine the sequence of Gap 1 and Gap 2, we mapped the raw reads of the *SB* PacBio whole genome sequencing onto Si_gnH.scaffold00042, and found four reads that crossed Gap 1 (three reads) and Gap 2 (one read) (table S7). Since the error rate of PacBio sequence is about 15% (Korlach 2015; Rhoads and Au 2015), we examined if the PacBio reads corresponded to any Si_gnH scaffolds. In this way, we found that Si_gnH.contig28264 corresponded to Gap 1, and thus, we filled Gap 1 with Si_gnH.contig28264. We subsequently scaffolded BigBt4p 001445F and BigBt4p 000408F using the post-PacBio gap-filled Si_gnH.scaffold00042, and truncated the sequence to a 100 kb contig, which we named BigB_100kb_at_OBP12b'_insertion.

In the *S.geminata* PacBio genome (version t3p), the flanking regions that surround *SiOBP12b'* are located on contig 000075F

We then aligned candidate regions and visualized with Mauve (Darling et al. 2004).

Rapid Amplification of cDNA Ends (RACE) assays

To determine if the *SiOBP12B* or *SiOBP12b* alleles or *SiOBP12b'* paralog was expressed in the antennae samples, we conducted 5' and 3' RACE using one of the P_Bb RNA samples that was used for the OBP qRT-PCR assays. RACE primers are provided in table S6.

3' RACE: We used 3'-RACE CDS Primer A from the SMART RACE cDNA Amplification Kit (634914, Ambion) to reverse transcribe 1st strand cDNA. Subsequently, the *SiOBP12* cDNA was amplified in a nested fashion with two amplification rounds. The 1st round was done using the primer OBP12-F227 and the Long Universal Primer A. The 2nd round was done with the OBP12-R302R (the reverse complement sequence of the qRT-PCR primer OBP12-R302) and the Short Universal Primer A.

5' RACE: We used SuperScript III (18080044, Invitrogen) and anchored oligo-dT to reverse transcribe 1st strand cDNA. Next we degraded the RNA using a mixture of RNaseH (18021014, Invitrogen) and RNaseA (AM2270, Invitrogen) (3:1) at 37°C for 30 minutes. Subsequently, we purified the 1st strand cDNA by ethanol precipitation followed by polyC-tailing using TdT enzyme (EP0161, Thermo Fisher). The 5' end of *SiOBP12* was amplified in a nested fashion with two amplification rounds using the gene specific primers and the primers provided in the 5′ RACE System V2.0 kit (18374-058, Invitrogen). The 1st round used the OBP12-R302R primer (the reverse complement sequence of the qRT-PCR primer OBP12-R302) and the 5' RACE Abridged Anchor Primer. The 2nd round used the OBP12-F227 and the 5' RACE Abridge Universal Anchor Primer.

Both 3' RACE and 5' end of *SiOBP12* were gel purified to select different product sizes. The purified PCR products were cloned and sequenced (Genomics BioScience & Technology Ltd., Taipei, Taiwan)).

To confirm the existence of *SiOBP12b'*, we used the primers that were designed on the 5' and 3' UTR based on the 5' and 3' RACE results (table S6, *SiOBP12b'* NCBI MN193778) to amplify the gene. We subsequently cloned the PCR products into a plasmid using the Zero Blunt TOPO PCR Cloning kit (450245, Invitrogen) following the manufacturer's instructions, and sequenced the insert from both ends with M13F(-20) and M13R(-24) primers.

Expression of *SiOBP12b'* and *SiOBP12B* in different worker body parts using a Sanger sequencing trace profile assay

To detect the expression differences between *SiOBP12B* and *SiOBP12b',* we used Sanger sequencing trace profiles, which has been used to show allele specific expression in other studies, such as (Gensterblum-Miller et al. 2018; Valente et al. 2019; Yordanova et al. 2019). To test whether we can use Sanger sequencing trace profiles to detect differences in the expression of *SiOBP12b'* and *SiOBP12B*, we constructed plasmids (QIAgen Plasmid Purification kit, QIAgen) containing the *SiOBP12b'* or *SiOBP12B* cDNA. The *SiOBP12b'* plasmid was obtained from RACE assays (above). To obtain *SiOBP12B* cDNA, we extracted total RNA from whole body of an *SB/SB* worker of a monogyne colony, and amplified the gene. We subsequently purified the PCR product and cloned into the TOPO-TA vector and verified by sequencing. Plasmid concentrations were measured by the Qubit dsDNA BR Assay kit (Q32853, Thermo Fisher) and the two plasmids diluted to the same stock concentration. We then sequenced four mixtures of *SiOBP12B* and *SiOBP12b'* containing plasmids at different ratios (*SiOBP12B*/*SiOBP12b'*): 1:1, 10:1, 1:10, and 1:4 (sequencing trace profiles in Dryad). The sequence trace profiles of these mixtures showed that different gene ratios can be observed, although the fold difference is not precise.

We conducted three bio-replicates of sequence trace profiles on the polygyne *SB/Sb* worker antennae, heads, and bodies (figure S1C). We amplified *SiOBP12B* and *SiOBP12b'* using the OBP12Bb*-3'UTR and the OBP12Bb*-5'UTR primers. The PCR products were sequenced using the OBP12-F227R primer without purification.

PCR purification, plasmid constructions, and cloning

To prepare fresh PCR products for cloning, the PCR products were subjected to either PCR purification (QIAquick PCR Purification kit, QIAgen) or gel purification (QIAquick Gel Extraction kit, QIAgen) following the manufacturer's instructions.

All the cloning reactions were conducted with either the Zero Blunt TOPO PCR Cloning kit (450245, Invitrogen) or the TOPO TA Cloning kit (450641, Invitrogen), depending on the PCR polymerase used in the amplification step. The former kit was used if the PCR assays were conducted with high fidelity polymerase KAPA HiFi Hotstart ReadyMix PCR kit (KK2601, KAPABiosystems) while the latter kit was used for all other PCR polymerases. Cloning followed the manufacturer's instructions with the maximum volume of PCR product input (4 μl).

Plasmids were transformed into the *E. coli* strain DH5-α.

REFERENCES

Altschul SF, Gish W, Miller W, Myers EW, Lipman DJ. 1990. Basic Local Alignment Search Tool. *J. Mol. Biol.* [Internet] 215(3):403–410. Available from: http://dx.doi.org/10.1016/S0022-2836(05)80360-2

Van der Auwera GA, Carneiro MO, Hartl C, Poplin R, Del Angel G, Levy-Moonshine A, Jordan T, Shakir K, Roazen D, Thibault J, et al. 2002. From FastQ data to high confidence variant calls: the Genome Analysis Toolkit best practices pipeline. *Curr. Protoc. Bioinforma.* 11(1110).

Bethesda (MD): National Library of Medicine (US) NC for BI. National Center for Biotechnology Information (NCBI). Available from: https://www.ncbi.nlm.nih.gov/

Darling ACE, Mau B, Blattner FR, Perna NT. 2004. Mauve: Multiple alignment of conserved genomic sequence with rearrangements. *Genome Res.* 14(7):1394–1403.

DePristo MA, Banks E, Poplin R, Garimella K V, Maguire JR, Hartl C, Philippakis AA, del Angel G, Rivas MA, Hanna M, et al. 2011. A framework for variation discovery and genotyping using next-generation DNA sequencing data. *Nat. Genet.* 43(5):491–498.

Fontana S, Chang N-C, Chang T, Lee C-C, Dang V-D, Wang J. The fire ant social supergene is characterized by extensive gene and transposable element copy number variation (in review).

Fu L, Niu B, Zhu Z, Wu S, Li W. 2012. CD-HIT: accelerated for clustering the next-generation sequencing data. *Bioinformatics* [Internet] 28(23):3150–3152. Available from: https://academic.oup.com/bioinformatics/article-lookup/doi/10.1093/bioinformatics/bts565

Gensterblum-Miller E, Wu W, Sawalha AH. 2018. Novel Transcriptional Activity and Extensive Allelic Imbalance in the Human MHC Region. *J. Immunol.* [Internet] 200(4):1496–1503. Available from: http://www.ncbi.nlm.nih.gov/pubmed/29311362

Gotzek D, Robertson HM, Wurm Y, Shoemaker D. 2011. Odorant binding proteins of the red imported fire ant, *Solenopsis invicta*: An example of the problems facing the analysis of widely divergent proteins. *PLoS One* 6(1):e16289.

Gotzek D, Ross KG. 2008. Experimental conversion of colony social organization in fire ants (*Solenopsis invicta*): Worker genotype manipulation in the absence of queen effects. *J. Insect Behav.* 21(5):337–350.

Huang Y, Dang VD, Chang N, Wang J. 2018. Multiple large inversions and breakpoint rewiring of gene expression in the evolution of the fire ant social supergene. *Proc. R. Soc. B* 285(1878):20180221.

Jouvenaz DP, Allen GE, Banks WA, Wojcik DP. 1977. A survey for pathogens of fire ants, *Solenopsis* spp., in the Southeastern United States. *Florida Entomol.* 60(4):275–279.

Korlach J. 2015. Understanding accuracy in SMRT sequencing. Available from: https://www.pacb.com/wp-content/uploads/2015/09/Perspective_UnderstandingAccuracySMRTSequencing.pdf

Krieger MJB, Ross KG. 2002. Identification of a major gene regulating complex social behavior. *Science (80-.).* 295(5553):328–332.

Krieger MJB, Ross KG. 2005. Molecular evolutionary analyses of the odorant-binding protein gene Gp-9 in fire ants and other Solenopsis species. *Mol. Biol. Evol.* 22(10):2090–2103.

Kumar S, Stecher G, Tamura K. 2016. MEGA7: Molecular Evolutionary Genetics Analysis version 7.0 for bigger datasets. *Mol. Biol. Evol.* [Internet] 33(7):1870–1874. Available from: https://academic.oup.com/mbe/article-lookup/doi/10.1093/molbev/msw054

Langmead B, Salzberg SL. 2012. Fast gapped-read alignment with Bowtie 2. *Nat. Methods* [Internet] 9(4):357–359. Available from: http://www.nature.com/articles/nmeth.1923

Langmead B, Wilks C, Antonescu V, Charles R. 2019. Scaling read aligners to hundreds of threads on general-purpose processors.Hancock J, editor. *Bioinformatics* [Internet] 35(3):421–432. Available from: https://academic.oup.com/bioinformatics/article/35/3/421/5055585

Leng N, Dawson JA, Thomson JA, Ruotti V, Rissman AI, Smits BMG, Haag JD, Gould MN, Stewart RM, Kendziorski C. 2013. EBSeq: An empirical Bayes hierarchical model for inference in RNA-seq experiments. *Bioinformatics* 29(8):1035–1043.

Li B, Dewey CN. 2011. RSEM: accurate transcript quantification from RNA-Seq data with or without a reference genome. *BMC Bioinformatics* 12(1):323.

Li H, Durbin R. 2009. Fast and accurate short read alignment with Burrows–Wheeler transform. *Bioinformatics* 25(14):1754–1760.

Li W, Godzik A. 2006. Cd-hit: a fast program for clustering and comparing large sets of protein or nucleotide sequences. *Bioinformatics* [Internet] 22(13):1658–1659. Available from: https://academic.oup.com/bioinformatics/article-lookup/doi/10.1093/bioinformatics/btl158

Martin M. 2011. Cutadapt removes adapter sequences from high-throughput sequencing reads. *EMBnet.journal* 17(1):10–12.

McKenna A, Hanna M, Banks E, Sivachenko A, Cibulskis K, Kernytsky A, Garimella K, Altshuler D, Gabriel S, Daly M, et al. 2010. The genome analysis toolkit: A MapReduce framework for analyzing next-generation DNA sequencing data. *Genome Res.* 20(9):1297–1303.

Pracana R, Priyam A, Levantis I, Nichols RA, Wurm Y. 2017. The fire ant social chromosome supergene variant *Sb* shows low diversity but high divergence from *SB*. *Mol. Ecol.* 26(11):2864–2879.

Premier Biosoft International. Beacon Designer. Available from: http://www.premierbiosoft.com/qOligo/Oligo.jsp?PID=1

Privman E, Cohen P, Cohanim AB, Riba-Grognuz O, Shoemaker D, Keller L. 2018. Positive selection on sociobiological traits in invasive fire ants. *Mol. Ecol.*:0–2.

Rhoads A, Au KF. 2015. PacBio Sequencing and its applications. *Genomics, Proteomics Bioinforma.* [Internet] 13(5):278–289. Available from: https://www.sciencedirect.com/science/article/pii/S1672022915001345

Sandler BH, Nikonova L, Leal WS, Clardy J. 2000. Sexual attraction in the silkworm moth: structure of the pheromone-binding-protein-bombykol complex. *Chem. Biol.* 7(2):143–151.

Shanker S, Paulson A, Edenberg HJ, Peak A, Perera A, Alekseyev YO, Beckloff N, Bivens NJ, Donnelly R, Gillaspy AF, et al. 2015. Evaluation of commercially available RNA amplification kits for RNA sequencing using very low input amounts of total RNA. *J. Biomol. Tech.* 26(1):4–18.

Song Y, Milon B, Ott S, Zhao X, Sadzewicz L, Shetty A, Boger ET, Tallon LJ, Morell RJ, Mahurkar A, et al. 2018. A comparative analysis of library prep approaches for sequencing low input translatome samples. *BMC Genomics* 19(1):1–16.

Sun Z, Asmann YW, Nair A, Zhang Y, Wang L, Kalari KR, Bhagwate A V., Baker TR, Carr JM, Kocher J-PA, et al. 2013. Impact of library preparation on downstream analysis and interpretation of RNA-Seq data: comparison between Illumina PolyA and NuGEN Ovation protocol. *PLoS One* 8(8):e71745.

Thornton B, Basu C. 2011. Real-time PCR (qPCR) primer design using free online software. *Biochem. Mol. Biol. Educ.* 39(2):145–154.

Trapnell C, Roberts A, Goff L, Pertea G, Kim D, Kelley DR, Pimentel H, Salzberg SL, Rinn JL, Pachter L. 2012. Differential gene and transcript expression analysis of RNA-seq experiments with TopHat and Cufflinks. *Nat. Protoc.* 7(3):562–578.

Valente FM, Sparago A, Freschi A, Hill-Harfe K, Maas SM, Frints SGM, Alders M, Pignata L, Franzese M, Angelini C, et al. 2019. Transcription alterations of KCNQ1 associated with imprinted methylation defects in the Beckwith–Wiedemann locus. *Genet. Med.* [Internet] 21(8):1808–1820. Available from: http://www.nature.com/articles/s41436-018-0416-7

Wang J, Jemielity S, Uva P, Wurm Y, Gräff J, Keller L. 2007. An annotated cDNA library and microarray for large-scale gene-expression studies in the ant *Solenopsis invicta*. *Genome Biol.* 8(1):R9.

Wang J, Wurm Y, Nipitwattanaphon M, Riba-Grognuz O, Huang Y-C, Shoemaker D, Keller L. 2013. A Y-like social chromosome causes alternative colony organization in fire ants. *Nature* 493(7434):664–668.

Wurm Y, Uva P, Ricci F, Wang J, Jemielity S, Iseli C, Falquet L, Keller L. 2009. Fourmidable: a database for ant genomics. *BMC Genomics* 10(1):5.

Wurm Y, Wang J, Riba-Grognuz O, Corona M, Nygaard S, Hunt BG, Ingram KK, Falquet L, Nipitwattanaphon M, Gotzek D, et al. 2011. The genome of the fire ant *Solenopsis invicta*. *Proc. Natl. Acad. Sci.* 108(14):5679–5684.

Xie C, Tammi MT. 2009. CNV-seq, a new method to detect copy number variation using high-throughput sequencing. *BMC Bioinformatics*.

Yordanova I, Pavlova Z, Kirov A, Todorov T, Alexiev A, Sarafov S, Mateva L, Chamova T, Gospodinova M, Mitev V, et al. 2019. Monoallelic expression of the TTR gene as a contributor to the age at onset and penetrance of TTR-related amyloidosis. *Gene* [Internet] 705:16–21. Available from: https://www.sciencedirect.com/science/article/abs/pii/S0378111919303816

Zhang H, Finiguerra M, Dam HG, Huang Y, Xu D, Liu G, Lin S. 2013. An improved method for achieving high-quality RNA for copepod transcriptomic studies. *J. Exp. Mar. Bio. Ecol.* 446:57–66.

Zuker M. 2003. Mfold web server for nucleic acid folding and hybridization prediction. *Nucleic Acids Res.* [Internet] 31(13):3406–3415. Available from: http://www.ncbi.nlm.nih.gov/pubmed/12824337
